# Understanding system dynamics of an adaptive enzyme network from globally profiled kinetic parameters

**DOI:** 10.1186/1752-0509-8-4

**Published:** 2014-01-15

**Authors:** Austin WT Chiang, Wei-Chung Liu, Pep Charusanti, Ming-Jing Hwang

**Affiliations:** 1 Institute of BioMedical Informatics, National Yang-Ming University, Taipei, Taiwan; 2 Bioinformatics Program, Taiwan International Graduate Program, Institute of Information Science, Academia Sinica, Taipei, Taiwan; 3 Institute of Biomedical Sciences, Academia Sinica, Taipei, Taiwan; 4 Institute of Statistical Science, Academia Sinica, Taipei, Taiwan; 5 Department of Bioengineering, University of California, San Diego, La Jolla, CA, USA

**Keywords:** Kinetic motif, Parameter profile, Biological network, Systems biology

## Abstract

**Background:**

A major challenge in mathematical modeling of biological systems is to determine how model parameters contribute to systems dynamics. As biological processes are often complex in nature, it is desirable to address this issue using a systematic approach. Here, we propose a simple methodology that first performs an enrichment test to find patterns in the values of globally profiled kinetic parameters with which a model can produce the required system dynamics; this is then followed by a statistical test to elucidate the association between individual parameters and different parts of the system’s dynamics.

**Results:**

We demonstrate our methodology on a prototype biological system of perfect adaptation dynamics, namely the chemotaxis model for *Escherichia coli*. Our results agreed well with those derived from experimental data and theoretical studies in the literature. Using this model system, we showed that there are motifs in kinetic parameters and that these motifs are governed by constraints of the specified system dynamics.

**Conclusions:**

A systematic approach based on enrichment statistical tests has been developed to elucidate the relationships between model parameters and the roles they play in affecting system dynamics of a prototype biological network. The proposed approach is generally applicable and therefore can find wide use in systems biology modeling research.

## Background

Systems biology aims to unravel the design principles of living organisms from a network perspective [1,2]. Advances in experimental studies have generated a large amount of data on several key biological processes [3-8], and networks of interactions between molecular species have been hypothesized [9-14]. Despite these advances, one unresolved challenge in systems biology is to understand how the hypothesized molecular interactions can lead to the observed biological phenomenon for complex biological systems. One way of pursuing this is via mathematical modeling of biological processes, which can also generate testable hypothesis for future experiments.

Biological processes are often complicated, and the complexity of their mathematical models usually increases with the amount of parameters involved. This generally gives rise to two fundamental problems in mathematical modeling. First, it is possible to have multiple sets of parameter values that are equally likely to produce the observed data and finding the “best” parameter set might be insufficient to fully characterize a biological system if such a parameter set is not the only set with biological relevance [15]. Second, understanding the role of individual parameters on different aspects of observed systems dynamics can be difficult for parameter-rich models as it might be too time consuming to explore this systematically and exhaustively. Although a number of approaches, for instance the genetic algorithm-based method [16] and others [15,17], have been developed to search for parameter solutions in high dimensional spaces, they have not been extended to make inferences on the contribution of individual parameters to specific components of the system dynamics. As such, in this paper we propose a general framework for addressing the aforementioned problems in mathematical modeling of biological systems. Our methodology can be summarized as follows. For a given mathematical model of a biological process, one first defines the system’s dynamics that the model is required to reproduce; one then searches through the parameter space by a sampling method, and keeps those sets of parameters with which the model can produce the required dynamics. These then form a matrix ***Z*** of functional parameter values, with each element *z*_*ij*_ denoting the value of the *j-*th parameter in the *i*-th parameter set. This matrix then undergoes statistical analysis to test whether a particular parameter is biased towards a certain value (or certain range of values) for the model to produce the target dynamics. After this is done for all parameters, the results can be compiled to identify recurrent parameter values and any patterns they might form. Finally, if the system’s dynamics can be decomposed into different components or parts, then further analysis can be performed to associate a parameter with a particular aspect of the dynamics.

**Figure 1:**
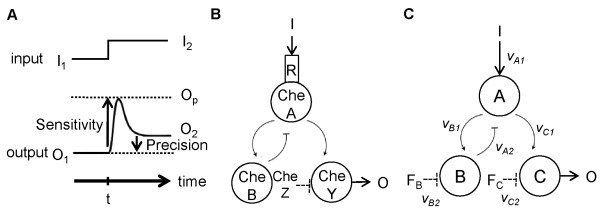
**Dynamics and models of an adaptive enzyme network. (A)** A schematic illustration of adaptation dynamics: sensitivity refers to the magnitude of the change in the output after the introduction of an external signal, and precision refers to the ability of the system returning to its pre-stimulus state after being perturbed by the external signal. Two quantities, a sensitivity score and a precision score, can be defined to measure these two dynamic properties (see Methods for details). **(B)** Network topology of the chemotaxis machinery in *E. coli*. **(C)** An enzyme network with a negative feed-back loop, known as the NFBLB model, that exhibits similar topology to the *E. coli* chemotaxis circuit where *v*_*n1*_ (*v*_*n2*_) (n = *A*, *B* or *C*) represents the activation (deactivation) process of the rate equation for node *n*.

Our methodology is demonstrated on a well-studied example of adaptation dynamics, that of the chemotaxis of *Escherichia coli*[18-21]. Generally speaking, adaptation refers to the ability of an organism adjusting to a new environment, and it is thought to be an important attribute for survival under fluctuating conditions [22-28]. In *Escherichia coli*, adaptation allows its chemotaxis system to reset stimulus-induced output to pre-stimulus value, even upon persistent external stimulation [29,30]. The dynamics of chemotaxis adaptation has two parts (Figure 1A): first, the output signal of the system exhibits a sharp increase after the initial stimulation, and this is referred to as the sensitivity phase; second, after the initial sharp rise, the output signal decays to its initial state, and this is referred to as the precision phase. Figure 1B illustrates the molecular processes involved, which have been identified experimentally [31,32]. Briefly, input signals are fed into the histidine kinase CheA-chemoreceptor R complex, and CheA then phosphorylates CheY (which can be dephosphorylated by CheZ) to regulate the process that drives the flagella of a bacteria. The key point here is that the activity of CheA is determined by the level of methylation of the CheA-bound receptor complex, which is controlled by demethylase CheB, which is in turn positively regulated by CheA itself, forming a negative feedback loop (Figure 1B). Recently, Ma et al. [33] constructed a mathematical model for a three-node enzyme network and found that only two major types of network topologies can produce dynamics associated with adaptation (Figure 1C and Additional file 1: Figure S8). One topology consists of a negative feed-back loop with a buffer node (NFBLB for short): node *A* positively influences the production of nodes *B* and *C*, and node *B* in return negatively regulates node *A* (Figure 1C). The other topology has an incoherent feed-forward loop with a proportioner node (IFFLP for short): here, node *A* induces node *B*, which in turn induces node *C*, and nodes *A*, *B* and *C* also have inhibiting role on nodes *C*, *A* and *B*, respectively (Additional file 1: Figure S8). The enzyme network driving the chemotaxis of *E. coli* has been found to resemble the NFBLB model [33]. Thus, in the rest of this paper, we use the NFBLB model to demonstrate our methodology in order to better understand how individual parameters contribute to the mechanism underlying the chemotaxis of *E. coli*. Empirical findings from the literature will be compared to our numerical results for validation purposes. Results for the IFFLP model will be presented in supplemental data.

## Methods

### Model of the adaptive enzyme network

The original *E. coli* chemotaxis model was proposed as a two-state model [29] and was later expanded to include the phosphorylation cascade [34]. In this study, the model we used is essentially the same as that used by [29] and [34], in which we consider the CheA-bound receptor complex as a single entity (node A in Figure 1C) and assume that this receptor complex exists in either a CheA active (*A*^*m*^) or a CheA inactive (*A*) state. The superscript *m* denotes the methylated form of the receptor complex.

The binding kinetic equation for the inactive receptor complex is given by,

(1) $$I+A\overset{K_{\mathrm{IA}}}{↔}\mathrm{IA}\overset{k_{\mathrm{IA}}}{\to }I+A^{m},$$

where *I* denotes the concentration of chemo-attractant, *K*_*IA*_ is the ligand dissociation constant and *k*_*IA*_ is the ligand catalytic constant.

The demethylation kinetic equation for the active receptor complex is given by,

(2) $$B^{P}+A^{m}\overset{K_{\mathrm{BA}}}{↔}B^{P}A^{m}\overset{k_{\mathrm{BA}}}{\to }B^{P}+A,$$

where *B*^*P*^ denotes the concentration of phosphorylated demethylase CheB, *K*_*BA*_ is the dissociation constant of phosphorylated CheB and *k*_*BA*_ is the catalytic constant of phosphorylated CheB.

The process of phosphate group transfer from active CheA to CheB is given by,

(3) $$A^{m}+B\overset{K_{\mathrm{AB}}}{↔}A^{m}B\overset{k_{\mathrm{AB}}}{\to }A^{m}+B^{P},$$

where *K*_*AB*_ and *k*_*AB*_ are the dissociation constant and the catalytic constant of active CheA, respectively.

The dephosphorylation of phosphorylated CheB is given by,

(4) $$B^{P}\overset{k_{F_{B}B}}{\to }B,$$

where *k*_*FBB*_ is the dephosphorylation constant.

Transfer of the phosphate group from active CheA to CheY (node C in Figure 1C) is given by,

(5) $$A^{m}+C\overset{K_{\mathrm{AC}}}{↔}A^{m}C\overset{k_{\mathrm{AC}}}{\to }A^{m}+C^{P},$$

where *K*_*AC*_ and *k*_*AC*_ are the dissociation constant and the catalytic constant of active CheA, respectively.

The dephosphorylation of phosphorylated CheY by CheZ (represented by *F*_*C*_ in Figure 1C and by Z in the equation below) is given by,

(6) $$Z+C^{P}\overset{K_{\mathrm{ZC}}}{↔}ZC^{P}\overset{k_{\mathrm{ZC}}}{\to }Z+C,$$

where *K*_*ZC*_ and *k*_*ZC*_ are the dissociation constant and the catalytic constant of CheZ, respectively.

The dynamics of these processes can be described by using a set of differential equations that model the NFBLB network depicted in Figure 1C [33]:

(7) $$\begin{array}{l} \frac{\mathrm{dA}}{\mathrm{dt}}=\underset{v_{A1}}{\underset{︸}{Ik_{\mathrm{IA}}\frac{\left(1-A\right)}{\left(1-A\right)+K_{\mathrm{IA}}}}}-\underset{v_{A2}}{\underset{︸}{Bk_{\mathrm{BA}}\frac{A}{A+K_{\mathrm{BA}}}}}\\ \frac{\mathrm{dB}}{\mathrm{dt}}=\underset{v_{B1}}{\underset{︸}{Ak_{\mathrm{AB}}\frac{\left(1-B\right)}{\left(1-B\right)+K_{\mathrm{AB}}}}}-\underset{v_{B2}}{\underset{︸}{F_{B}k_{F_{B}B}\frac{B}{B+K_{F_{B}B}}}}\\ \frac{\mathrm{dC}}{\mathrm{dt}}=\underset{v_{C1}}{\underset{︸}{Ak_{\mathrm{AC}}\frac{\left(1-C\right)}{\left(1-C\right)+K_{\mathrm{AC}}}}}-\underset{v_{C2}}{\underset{︸}{F_{C}k_{F_{C}C}\frac{C}{C+K_{F_{C}C}}}} \end{array}$$

where *I* denotes the input signal (i.e. the concentration of chemo-attractant); *A*, *B* and *C* denote the concentrations in active states; (1-*A*), (1-*B*) and (1-*C*) denote the concentrations in inactive forms; *F*_*B*_ and *F*_*C*_ are the concentration of deactivating enzymes (assumed to be a constant value of 0.5 as in [33]) that transform the active states of *B* and *C* into inactive states. Kinetic parameters *k*_*ij*_ and *K*_*ij*_ are respectively the catalytic rate constant and Michaelis-Menten constant for the catalytic reaction between substrate *i* and its regulator (activating or deactivating) enzyme *j*, where *i* and *j* = *A*, *B*, *C*, *F*_*B*_, or *F*_*C*_. Note that for each node *A*, *B* or *C*, the first term (*v*_*1*_) and the second term (*v*_*2*_) of the equation represent its activation and deactivation rates respectively.

### Measuring adaptation

Two quantities were used to evaluate the performance of a kinetic parameter set in producing adaptation dynamics: (i) sensitivity to the input stimulus (equation (8)), which is defined as the difference between output response and the initial steady-state value, and (ii) precision (equation (9)), which is defined as the inverse of the difference between pre- and post-stimulus steady state values. The corresponding mathematical equations for these two quantities are [33]:

(8) $$\mathrm{Sensitivity}=\frac{\left|O_{p}-O_{1}\right|/O_{1}}{\left|I_{2}-I_{1}\right|/I_{1}}$$

(9) $$\mathrm{Precision}={\left(\frac{\left|O_{2}-O_{1}\right|/O_{1}}{\left|I_{2}-I_{1}\right|/I_{1}}\right)}^{-1}$$

where O_1_ and O_2_ represent two steady-state values, respectively corresponding to the two input values I_1_ and I_2_ (I_1_ = 0.5 and I_2_ =0.6 following [33]), and O_p_ is the peak value of a transient pulse in response to the input change (see Figure 1A).

### Sampling parameter values and numerical simulations

As in [33], Latin hypercube sampling [35] was used to sample uniformly at random the values of kinetic parameters on a logarithmic scale, with the catalytic rate constant *k* being in the range of [10^-1^, 10^1^] and the Michaelis-Menten constant *K* in the range of [10^-3^, 10^2^]. These two parameter ranges were chosen by Ma et al. [33] to, presumably, minimize computational cost while covering previously reported values used to model the *E coli* chemotaxis system [36-38]. In order for our results to be comparable with those from previous studies, we opted to use the same parameter ranges in this paper. For each parameter set, the model (equation (7) for the NFBLB model and equation (S1) in the supplemental data for the IFFLP model) was numerically simulated and those producing the desired adaptation dynamics were identified. The original 10^4^ parameter sets sampled by Ma *et al.* for the NFBLB model produced only eight kinetic solutions [33], which are insufficient for discovering parameter motifs with any statistical significance. To remedy this, a 10-fold greater sampling size was used in this study; for the IFFLP model, the original 10^4^ sampling produced 131 solutions, enough for the subsequent enrichment tests to be carried out. Results from additional sets of simulation and also from a run increasing the sample size to 10 fold, which increased the number of kinetic solutions to roughly 10 fold, indicated that the parameter motifs reported here had been reliably deduced (see Discussion).

Following Ma et al. [33], we discarded those parameter sets that render the model to produce extremely small steady-state values, persistent oscillations, weakly damped oscillations, and exceedingly long transient dynamics. For each of the remaining parameter sets (46,715 for NFBLB and 6,073 for IFFLP), the sensitivity and precision scores (i.e. equation (8) and equation (9) respectively) were calculated. We said a particular parameter set was a kinetic solution to the model if its sensitivity score was greater than 1 and its precision score greater than 10, as these criteria have been used to define perfect adaptation [33]. It can be shown from equation (8) and (9) that these thresholds were chosen to ensure a stimulus of at least 20% of the initial steady-state value, and the system can return back to this value within an error of 2%, in consistence with those used in experimental measurements (Khan et al. [39] and Alon et al. [40]). More stringent thresholds reduced the value range of the parameter motifs, but the resultant minor changes did not significantly affect the main findings (see Discussion). We used computer programs from Ma et al. [33] and Matlab software (version 7.6.0.324, release R2008a) [41] available at http://www.mathworks.com, to implement a computational pipeline to simulate both the NFBLB model and the IFFLP model numerically. We validated our simulation pipeline by reproducing the numerical results of [33] and also the steady-state solution of a four-node transcriptional regulation cascade of [42].

### Enrichment test

In order to see if there exists an underlying pattern in the values of kinetic parameters for the NFBLB model to yield perfect adaptation dynamics, we obtained the kinetic solutions and plotted the distribution of parameter values for each kinetic parameter. To test whether the resulting distributions of parameter values were enriched in the sets of kinetic solutions among those 10^5^ (NFBLB) parameter sets sampled, we adopted an enrichment test that has found many uses in genomics sciences [43,44]. For a given kinetic parameter, let *N* be the total number of parameter values sampled (here, *N* = 10^5^ for NFBLB model), and let *y*_*i*_ be the number of those in *N* belonging to the *i*^th^ value class in the logarithm scale (*i* = 1, 2, …, 5); furthermore, let *M* be the number of kinetic solutions (here, *M* = 74 for NFBLB model), and *x*_*i*_ be the number of those in *M* belonging to the same *i*^th^ value class. Parameter values belonging to the 1^st^ value class are within the interval [10^-3^, 10^-2^], those belonging to the 2^nd^ value class are within [10^-2^, 10^-1^], and so on and so forth, with the 5^th^ value class containing parameter values within [10^1^, 10^2^]. The five value classes of kinetic parameters may correspond to varying strengths of enzymatic reactions that can be measured and classified experimentally. Doubling the number of value classes resulted in a similar, albeit finer, map of parameter motifs, but did not alter the conclusions reached (see Discussion).

Under the condition of *M* being sampled independently and uniformly at random without replacement from *N*, the probability of observing *x*_*i*_ by chance follows a hypergeometric distribution [43,44]:

(10) $$p\left(n=x_{i}|y_{i},M,N\right)=\frac{\left(\begin{array}{c} y_{i}\\ x_{i} \end{array}\right)\left(\begin{array}{c} N-y_{i}\\ M-x_{i} \end{array}\right)}{\left(\begin{array}{c} N\\ M \end{array}\right)},$$

where *i* = 1, 2, 3, 4 and 5, and

$$\left(\begin{array}{c} N\\ M \end{array}\right)=\frac{N!}{M!\left(N-M\right)!}.$$

As in the enrichment test employed in genomics sciences [43,44], we can then compute the *p*-value to measure the statistical significance of the likelihood for observing *x*_*i*_ when the null distribution (equation (10)) is assumed to be the true count distribution. In this study, we used a *p*-value threshold of 10^-3^ to decide the statistical significance of the enrichment test.

Thus, for each Michaelis-Menten constant *K*, we carried out five independent enrichment tests, each for each value class, and for each catalytic rate constant *k*, we carried out two independent enrichment tests, one for the 3^rd^ value class (i.e. [10^-1^, 10^0^]) and the other for the 4^th^ value class (i.e. [10^0^, 10^1^]), due to the smaller range of parameter values for *k* (see above). If an enrichment test was statistically significant (*p*-value < 10^-3^), a motif in the form of value class was assigned to the kinetic parameter tested. To sum up, the outcome of this analysis produced motifs of kinetic parameters, which tells us whether a particular kinetic parameter is biased towards any specific value class on the logarithm scale, or none at all.

### Functional association test and parameter inter-dependence

For each kinetic parameter, values from all the kinetic parameter sets (46,715 for NFBLB and 6,073 for IFFLP) were partitioned into two groups. The first group, called the motif group, consists of parameter values belonging to the biased value class(es) as identified by the above mentioned enrichment test on kinetic solutions; and the second group, called the non-motif group, comprises those parameter values that are not in the motif group.

We then tested whether the motif group exhibited higher sensitivity or precision scores than the non-motif group by comparing the score medians of the two groups. Since the scores were not normally distributed, we used the Mann–Whitney *U*-Test [45] for the analysis. The test produced a *z*-score, and we said a particular kinetic parameter is positively associated with the sensitivity or precision parts of the adaptation dynamics if the corresponding *z*-score is greater than 3.29 (i.e. the upper boundary of the critical value of the 99.9% confidence intervals). In this study our focus was on finding parameters that can significantly improve the function, although some of the parameters may exhibit a large negative *z*-score indicative of a negative role in the function. A bipartite kinetic-functionality network can then be constructed to display the associations between the kinetic motifs and the functionalities identified.

Finally, we investigated the cooperation between every pair of kinetic parameters. Specifically, we took the kinetic parameters from those 74 kinetic solutions (131 for IFFLP) and performed Pearson correlation test between the values of a pair of kinetic parameters that appeared in the kinetic motifs identified. We said two parameters are correlated if the p-value of the correlation test is less than 0.05.

## Results

### Detecting kinetic motifs

**Figure 2:**
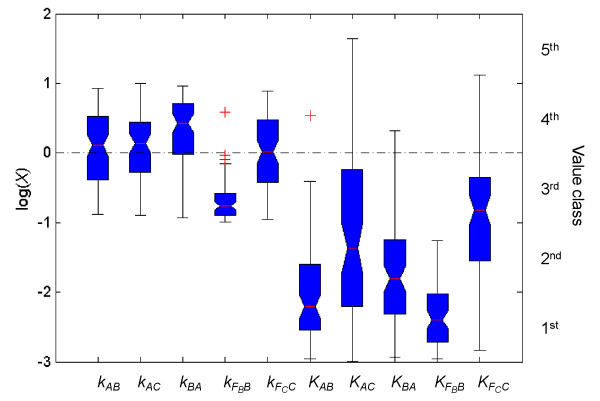
**Distributions of kinetic parameters.** Distributions of parameter values obtained from a total of 74 kinetic solutions exhibiting perfect adaptation for the NFBLB model. Each tick on the x axis is a specific catalytic rate constant *k* or Michaelis-Menten constant *K*, and the values of the parameters are in power of 10, which are divided into five value classes as indicated at the right of the figure. On each data box, the contracted center is the median, while the edges of the box are the 25^th^ and 75^th^ percentiles of the distribution.

As described in the Methods, we found only 74 sets of parameters from 10^5^ randomly sampled sets that could satisfy the criteria of perfect adaptation dynamics for the NFBLB model. Figure 2 shows the distributions of these 74 sets of parameter values. We can see that while some parameters, *k*_*FBB*_ especially, were limited to be within a relatively small range of values, others, like *k*_*FCC*_ and *K*_*AC*_, saw a distribution covering nearly the entire range of the values sampled. On the whole, catalytic rate constants *k*_*FBB*_ and *k*_*BA*_ were biased towards the 3^rd^ and the 4^th^ value class, respectively, while all five Michaelis-Menten constants were biased towards one or more of the first three value classes. Other catalytic rate constants, namely *k*_*AB*_, *k*_*AC*_ and *k*_*FCC*_, did not show apparent biases towards any vale classes. These observations were quantified with statistical significance by the enrichment tests; the results, summarized in Table 1, are consistent with the distributions of parameter values shown in Figure 2.

**Table 1:** Results of parameter enrichment test for the NFBLB model

***Parameter***	**1**^**st **^**class**			**2**^**nd **^**class**			**3**^**rd **^**class**			**4**^**th **^**class**			**5**^**th **^**class**		
	**[10**^**-3**^**, 10**^**-2**^**]**^***a***^			**[10**^**-2**^**, 10**^**-1**^**]**			**[10**^**-1**^**, 10**^**0**^**]**			**[10**^**0**^**, 10**^**1**^**]**			**[10**^**1**^**,10**^**2**^**]**		
	***x***^***b***^	***y***^***c***^	***p***^***d***^	***x***	***y***	***p***^***d***^	***x***	***y***	***p***^***d***^	***x***	***y***	***p***^***d***^	***x***	***y***	***p***^***d***^
***k***_***AB***_							32	49,999	8.5E-01	42	50,001	1.0E-01			
***k***_***AC***_							32	50,000	8.5E-01	42	50,000	1.0E-01			
***k***_***BA***_							24	50,000	1.0E + 00	50	50,000	**7.6E-04**			
***k***_***FBB***_							73	50,000	**<2.2E-16**	1	50,000	1.0E + 00			
***k***_***FCC***_							35	50,000	6.4E-01	39	50,000	2.8E-01			
***K***_***AB***_	44	19,998	**9.4E-11**	25	19,999	1.7E-03	4	20,000	1.0E + 00	1	20,000	1.0E + 00	0	20,000	1.0E + 00
***K***_***AC***_	26	20,000	**7.4E-04**	17	19,999	2.1E-01	17	20,000	2.1E-01	6	20,000	1.0E + 00	8	20,000	9.7E-01
***K***_***BA***_	33	19,994	**4.3E-07**	28	20,000	**1.1E-04**	11	20,000	8.3E-01	2	20,000	1.0E + 00	0	20,000	1.0E + 00
***K***_***FBB***_	57	19,997	**<2.2E-16**	17	19,999	2.1E-01	0	19,999	1.0E + 00	0	20,000	1.0E + 00	0	20,000	1.0E + 00
***K***_***FCC***_	9	19,999	9.4E-01	23	19,999	8.0E-03	32	20,000	**1.5E-06**	9	20,000	9.4E-01	1	20,000	1.0E + 00

^a^In square brackets are the intervals of parameter values for the indicated class.

^b^Out of *M* (=74) kinetic solutions, x is the number of solutions with the value of the indicated parameter belonging to the indicated value class.

^c^Out of a total of *N* (=10^5^) parameter sets sampled*,* y is the number of sets with the value of the indicated parameter belonging to the indicated value class.

^d^An enrichment test is considered statistically significant if its p-value < 10^-3^ and is highlighted in boldface. Based on *p*-values (using 10^-3^ as threshold), an enrichment state, i.e. motif, was assigned.

### Functional roles of kinetic motifs

For each of the seven kinetic parameters showing bias to at least one value class with statistical significance as determined in Table 1, we carried out Mann–Whitney *U*-test [45] to find out whether a motif (i.e. a preferred value class) of that parameter is involved in the sensitivity function of the adaptation dynamics, or, correspondingly, whether the median of the sensitivity scores for the motif group is significantly larger than that for the non-motif group (see Methods). The same analysis was then repeated for precision.

**Figure 3:**
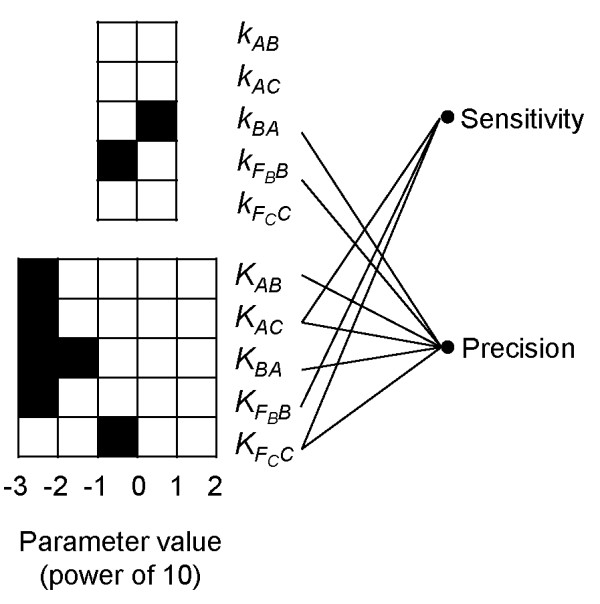
**A kinetic functionality network.** A bipartite network connecting kinetic parameters to functionalities (sensitivity and precision) of the adaptation dynamics. On the left are kinetic motifs emerged from the enrichment tests (see Methods), where filled boxes represent enriched values bounded by the indicated power of 10 for the indicated parameter. On the right are different functionalities (sensitivity and precision) of adaptation dynamics. A connection between a kinetic parameter and functionality was established if the association between the two was determined to be significant in the statistical test (see Methods).

The results, summarized in Table 2, indicate that three kinetic parameters, *K*_*AC*_, *K*_*FBB*_ and *K*_*FCC*_, were highly significant in improving sensitivity scores, and six kinetic parameters, *k*_*BA*_, *k*_*FBB*_, *K*_*AB*_, *K*_*AC*_, *K*_*BA*_ and *K*_*FCC*_, were highly significant in improving precision scores. Furthermore, for the majority of parameters, their motifs were either associated with improving sensitivity or precision scores, but not both. Two interesting exceptions are *K*_*AC*_ and *K*_*FCC*_ as their motifs tended to reflect improvements in both functionalities (Table 2). These observations are generally in accord with the results from an analytical analysis of the rate equations (see below and supplemental data). Note that sensitivity and precision are two conflicting dynamic processes with the former requiring the system to deviate from steady state abruptly and the latter requiring the system to return to the original steady state in a timely fashion (Figure 1A). Therefore, theoretically speaking, a parameter that improves one function will also have a negative effect on the other function, as reflected generally by the opposite signs of the z-scores obtained from the sensitivity and precision tests (Table 2). Finally, the above findings can be succinctly captured by drawing a kinetic-functionality network (Figure 3), which highlights both the functional roles and constraints (i.e. the enriched value classes) of kinetic parameters.

**Table 2:** Functional association test results for the NFBLB model

**Parameter**	**Motif [m]**^**a**^	**Non-motif [~m]**	** Precision test**^**b**^			** Sensitivity test**^**b**^			**Function**^**c**^
			**Pr**_**m**_	**Pr**_**~m**_	**z**^**d**^	**Sn**_**m**_	**Sn**_**~m**_	**z**^**d**^	
*k*_*BA*_	[10^0^,10^1^]	[10^-1^,10^0^]	1.81	1.35	**30.88**	−1.18	−0.76	−8.75	PR
*k*_*FBB*_	[10^-1^,10^0^]	[10^0^,10^1^]	1.68	1.45	**16.71**	−1.16	−0.80	−6.67	PR
*K*_*AB*_	[10^-3^, 10^-2^]	[10^-2^,10^2^]	1.59	1.42	**6.60**	−1.40	−0.90	−6.65	PR
*K*_*AC*_	[10^-3^, 10^-2^]	[10^-2^,10^2^]	1.57	1.52	**9.23**	1.23	−1.38	**26.57**	PR,SN
*K*_*BA*_	[10^-3^, 10^-1^]	[10^-1^,10^2^]	1.76	1.29	**35.34**	−1.41	−0.68	−12.72	PR
*K*_*FBB*_	[10^-3^, 10^-2^]	[10^-2^,10^2^]	1.47	2.05	−34.32	−0.61	−1.06	**3.95**	SN
*K*_*FCC*_	[10^-1^, 10^0^]	[10^-3^, 10^-1^] and [10^0^, 10^2^]	1.63	1.34	**16.98**	3.30	−2.31	**28.37**	PR,SN

^a^In square brackets are the intervals of the parameter values, ’m’ is the motif group and ‘~m’ the non-motif group. Note that for the kinetic parameters (*k*_*AB*_, *k*_*AC*_, and *k*_*FCC*_) showing no apparent bias towards any value classes, the statistical tests were not conducted because their parameter values could not be partitioned into motif group and non-motif group (see Methods).

^b^“Pr_m_” (“Sn_m_”) is the mean logarithm value of precision (sensitivity) scores for the motif group, and “Pr_~m_” (“Sn_~m_”) is the same but for the non-motif group.

^c^“PR” (*“*SN”) indicates that the corresponding kinetic motif is statistically significant (z-score ≧3.29) in improving precision (sensitivity).

^d^z-score greater than 3.29 (99.9% confidence interval) is highlighted in boldface.

### Cooperation of kinetic parameters

**Figure 4:**
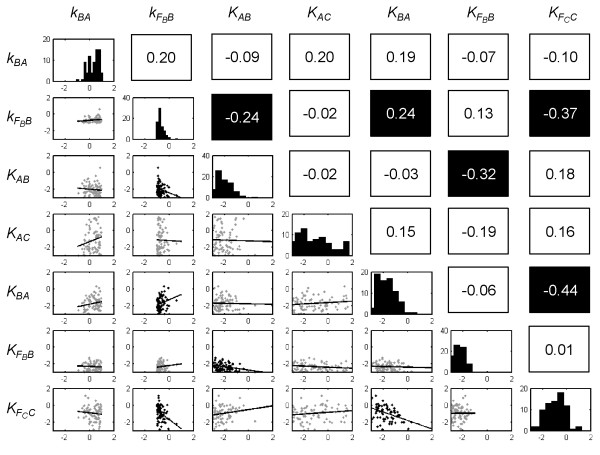
**Correlation between kinetic parameters.** The Pearson correlation coefficients between pairs of the seven parameters that exhibited value class enrichment are shown in the top-right triangle, where a box is colored black if the corresponding correlation is significant (p-value < 0.05). In the bottom left triangle, the scatter plots of the paired parameters are shown. On the diagonal are occurrence distributions of individual kinetic parameters.

Next, we asked the question of whether kinetic parameters work independently or in a cooperative way with each other when contributing to the system’s adaptation dynamics. Figure 4 shows the results of correlation tests performed on all pairs of the seven kinetic parameters that exhibited value class biases. We identified one significant positive correlation (*p-value* < 0.05) between parameters *k*_*FBB*_ and *K*_*BA*_, and four significant negative correlations in (*k*_*FBB*_, *K*_*AB*_), (*k*_*FBB*_, *K*_*FCC*_), (*K*_*AB*_, *K*_*FBB*_) and (*K*_*BA*_, *K*_*FCC*_) pairs. Interestingly, with the exception of *K*_*FBB*_, most of these correlated parameters contributed significantly to system’s precision (Figure 3), suggesting that they work in a cooperative manner in the precision mechanism of adaptation dynamics. In contrast, kinetic parameters contributing to the system’s sensitivity (i.e. *K*_*AC*_, *K*_*FBB*_ and *K*_*FCC*_, see Figure 3) were not correlated with each other, implying that they function independently in the sensitivity mechanism. If the mathematical model employed can indeed simulate the mechanics of adaptation in real biological systems, a corollary of these findings is that precision seems to be a more complicated mechanism than sensitivity in the system, thus the former requires many parameters to work together in order to achieve the desired level of high precision.

### Experimental data for kinetic parameters of *E. coli* chemotaxis

The NFBLB model is equivalent to the *E. coli* chemotaxis model with nodes *A*, *B* and *C* corresponding to the CheA-bound receptor complex, CheB and CheY, respectively. Chemotaxis of *E. coli* has been studied experimentally and parameters relating to individual processes involved in the model have been estimated [36-38,46]. Intriguingly, as described below, the kinetic motifs observed here for the NFBLB model compared well with the experimental data for the *E. coli* chemotaxis (Table 3).

**Table 3:** **Experimental data for the kinetic parameters of *E. coli *** **chemotaxis**

** Description**	** Reaction**^**a**^	**Eq7**^**b**^	***k*** **(s**^**-1**^**)**	***K***	**Notes**^**c **^**[Reference]**
Receptor Complex Demethylation	$B^{P}+A^{m}\overset{K_{\mathrm{BA}}}{↔}B^{P}A^{m}\overset{k_{\mathrm{BA}}}{\to }B^{P}+A$	*v*_*A2*_	1.2	0.08	*k*^*exp*^ = 1.2 s^-1^[46] and *K*^*exp*^ = 0.39 *μ*M [46]; *k*_*BA*_ = *k*^*exp*^ and *K*_*BA*_ *= K*^*exp*^/[A_t_].
CheB	$A^{m}+B\overset{K_{\mathrm{AB}}}{↔}A^{m}B\overset{k_{\mathrm{AB}}}{\to }A^{m}+B^{P}$	*v*_*B1*_	3.2	0.281	*k*^*exp*^ = 3.2 s^-1^[37] and *K*^*exp*^ = 1.405 *μ*M [37]; *k*_*AB*_ = *k*^*exp*^ and *K*_*AB*_ *= K*^*exp*^/[B_t_].
Phosphotransfer					
CheB	$B^{P}\overset{k_{F_{B}B}}{\to }B$	*v*_*B2*_	0.35	-	*k*^*exp*^ = 0.7 s^-1^ at 35°C, or 0.35 s^-1^ at 25°C [47]; *k*_*FBB*_ = *k*^*exp*^*.*
Dephosphotransfer					
CheY	$A^{m}+C\overset{K_{\mathrm{AC}}}{↔}A^{m}C\overset{k_{\mathrm{AC}}}{\to }A^{m}+C^{P}$	*v*_*C1*_	650	0.36	*k*^*exp*^ = 650 s^-1^[48] and *K*^*exp*^ = 6.5 *μ*M [48]; *k*_*AC*_ = *k*^*exp*^ and *K*_*AC*_ *= K*^*exp*^/[C_t_].
Phosphotransfer					
CheY	$F_{C}+C^{P}\overset{K_{F_{C}C}}{↔}F_{C}C^{P}\overset{k_{F_{C}C}}{\to }F_{C}+C$	*v*_*C2*_	30	0.006	*k*^*exp*^ = 650 s^-1^[34] and *K*^*exp*^ = 0.1 *μ*M [34]; *k*_*FCC*_ = *k*^*exp*^ and *K*_*FCC*_ *= K*^*exp*^/[C_t_].
Dephosphotransfer					

^a^Superscript *m* denotes methylated form and superscript *p* denotes phosphorylated form.

^b^Each biochemical reaction is equivalent to the process of activation (*v*_*n1*_) or deactivation (*v*_*n2*_) (*n* = *A*, *B*, and *C*) as indicated in the NFBLB model (see equation (7) in Methods).

^c^Total concentrations for the CheA-bound receptor complex ([A_t_]), CheB ([B_t_]) and CheY ([C_t_]) are 5.0 μM [34], 2.27 μM [37] and 17.9 μM [34], respectively. Superscript *exp* denotes experimental measurement.

To facilitate and simplify the comparison, we dictated that parameters with a value in the first three value classes (i.e., < 10^0^) are small-value parameters, while those in the 4^th^ and 5^th^ value class (i.e. ≧10^0^) are large-value parameters. Note that in enzyme kinetics, an enzyme with a large *K* value (Michaelis-Menten constant) indicates a weak binding affinity to its substrate, while a large *k* value (catalytic rate constant) implies the occurrence of a rapid catalytic event [49]. For sensitivity, the empirical estimates for *K*_*AC*_ and *K*_*FCC*_, after being normalized with the concentration of CheY, were 0.36 and 0.006 respectively (Table 3). This echoes our finding that both parameters should take small values. *K*_AC_ and *K*_*FCC*_ are respectively involved in the rates of phosphorylation (i.e. activation) and dephosphorylation (i.e. deactivation) of the response regulator CheY. Goldbeter and Koshland [50] explored a simple model of enzyme reaction and found that if the activating and deactivating enzymes operate at saturation where the substrate concentration does not affect reaction rate, then an ultra-sensitivity response is observed. Similar arguments may apply to chemotaxis in *E. coli*: namely, in order to produce sensitivity dynamics, both phosphorylating and dephosphorylating agents (i.e. CheA and CheZ, respectively) must be saturated, implying a situation where the concentration of the substrate (i.e. CheY) cannot alter the reaction rate. A close inspection of the rate reaction for node *C* in equation (7) suggests that both *K*_*AC*_ and *K*_*FCC*_ taking on small values can fulfil this condition. Note that if both *K*_*AC*_ and *K*_*FCC*_ are small, then *C*> > *K*_*FCC*_ and (1-*C*)> > *K*_*AC*_ such that

(11) $$\begin{array}{ll} \frac{\mathrm{dC}}{\mathrm{dt}} & =Ak_{\mathrm{AC}}\frac{\left(1-C\right)}{\left(1-C\right)+K_{\mathrm{AC}}}-F_{C}k_{F_{C}C}\frac{C}{C+K_{F_{C}C}}\\ & \approx Ak_{\mathrm{AC}}\frac{\left(1-C\right)}{\left(1-C\right)}-F_{C}k_{F_{C}C}\frac{C}{C}\\ & =Ak_{\mathrm{AC}}-F_{C}k_{F_{C}C} \end{array}$$

and the concentration of node *C* (i.e. CheY) disappears from the rate equation completely.

The kinetic parameter *K*_*FBB*_ is involved in the deactivation of node *B* in the NFBLB model and *K*_*FBB*_ is also found to be biased to small values (1^st^ value class motif in Table 1) for the model to exhibit sensitivity dynamics. Although node *B* of the NBFLP model corresponds to CheB in the chemotaxis of *E. coli*, to the best of our knowledge there are no natural enzymes reported to deactivate CheB^p^ in the manner suggested by the NFBLB model. Perhaps an unrevealed enzyme exists to dephosphorylate CheB^p^, the phosphorylate form of CheB, or there might be other mechanism of CheB^p^ dephosphorylation that is more complicated than the current knowledge can offer.

As for precision, the empirical estimates for *K*_*AB*_ (normalized with the concentration of CheB), *k*_*FBB*_, *K*_*BA*_ (normalized with the concentration of receptor complex) and *k*_*BA*_ are 0.281, 0.35, 0.08 and 1.2, respectively (Table 3). These are also in agreement with our findings (Table 1) that *K*_*AB*_, *k*_*FBB*_ and *K*_*BA*_ are biased towards small values, and *k*_*BA*_ is constrained to large values. *K*_*AB*_ and *k*_*FBB*_ are involved in the phosphorylation and dephosphorylation of CheB, respectively. According to Ma et al. [33], *K*_*AB*_ is constrained to small values by the topological features of the NFBLB model such that the rate equation of node *B* (i.e. CheB) is independent of the input level *I*; this then implies the system is in a stable state independent of the initial perturbation and thus is able to maintain high precision level. From equation (7), a small value for *k*_*FBB*_ can reduce the dephosphorylation rate of CheB, this in turn increases the deactivation rate of the receptor complex, thereby ensuing high precision. Finally, *K*_*BA*_ and *k*_*BA*_ play a part in the demethylation of the receptor complex, and intuitively the rate of its demethylation must be great (e.g. with a large *k*_*BA*_ and a small *K*_*BA*_) such that the perturbation initiated by the input signal can be mitigated in order to maintain high precision.

These observations of key parameters of the NFBLB model are furthermore supported by a number of experimental findings on the chemotaxis system of *E. coli*: 1) *K*_*FCC*_ having a large effect on the sensitivity part of the system dynamics is in agreement with CheZ playing an important role in adjusting the concentration of CheY [43]; 2) *K*_*AC*_ being an important kinetic parameter in affecting sensitivity agrees well with the receptor complex being a major contributor to sensitivity [44]; 3) CheB mutants being far less sensitive than the wild type due to the functional abnormality of the receptor complex in those mutants [45] implies that *K*_*FBB*_ is an important parameter affecting sensitivity; 4) that phosphorylated CheB can increase the rate of receptor demethylation and thus speeds up adaptation [51] supports the finding that *k*_*BA*_, *k*_*FBB*_, *K*_*AB*_ and *K*_*BA*_ were all important parameters in contributing to the system’s precision mechanism.

## Discussion

We have developed here a methodology using parameter profiling and enrichment statistical tests to uncover not only the sets of kinetic parameters with which a model produces user-specified system dynamics, but also motifs (i.e. enriched value classes) of these parameters and their associations with specific functional aspects of the system’s dynamics. For these tasks, conventional methods usually focus on identifying the “best” parameter set in fitting the empirical data and on using complicated analytical explorations of models (e.g., sensitivity analysis) [52-54] or by a laborious local approach examining how changing the parameters one at a time would affect systems dynamics [55-57]. Note that the sensitivity analysis of conventional methods (not to confuse with the sensitivity of adaptation (Equation 8) studied in this work) is usually carried out for one specific output dynamics, whereas our profiling approach investigated the sensitivity of functional elements (sensitivity and precision of adaptation) for a collection of outpout dynamics (those that qualified as a perfect adaptation; there were 74 for the NFBLB model). Interestingly, the kinetic parameters showing value class biases (Table 2) are those exhibiting non-negligible sensitivity indices obtained from analytical derivation (Additional file 1: Equation S2) or from the numerical method implemented in AMIGO [58] (Additional file 1: Figure S1), the latter two being computed using the output dynamics and the parameter set of a randomly chosen one of the 74 kinetic solutions. Note that bootstrap-derived distributions of the kinetic parameters and their confidence intervals for this particular kinetic solution were generally in accord with the kinetic motifs deduced from the 74 kinetic solutions (Additional file 1: Figure S2). In summary, by profiling the parameters as a whole our method takes a global view to find not just one but clusters of viable parameter sets, thus moving a step further to account for the complexity of biological systems. Although a number of global-view approaches have recently been developed to sample from large and high dimensional parameter spaces, including a combined global and local exploration [15] and an approach with model checking on partitioned regions of parameters [17], these studies do not make inferences on whether motifs of parameters exist and how they might contribute to specific elements of the system dynamics. Our approach therefore offers a simple framework to systematically characterize and elucidate the functional contribution of kinetic parameters in a biological network.

The kinetic motifs obtained are quite robust in that only minor differences in their resolution were resulted from independent sampling runs (Additional file 1: Figure S3), different thresholds of the adaptation objectives (Additional file 1: Figure S4), and added number of value classes (Additional file 1: Figure S5). Further analysis showed that, for NFBLB and to satisfy the statistical significance of p-value < 10^-3^, 8 × 10^4^ (rather than 10^5^) sampling runs were required to converge and stabilize the kinetic motifs (Additional file 1: Figure S6). The computational cost of our method is dominated by the sampling and simulation step (see an analysis Additional file 1: Section III). To investigate the difficulties that will inevitably arise from larger networks for our method, we artificially linked two modules of NFBLB together (see Additional file 1: section IV) while requiring the system to produce the same adaptation dynamics as before. The results showed that 5 times of sampling/simulations (4 × 10^5^ vs. 8 × 10^4^) were needed to produce stabilized kinetic motifs (Additional file 1: Figure S7) for the twice-sized network (20 kinetic parameters vs. 10). However, it should be noted that the required number of sampling/simulations depends on many factors, including the specified output dynamics, the level of statistical significance desired, and the network topology (e.g., the IFFLP model needed 10 times less number of sampling/simulations than the NFBLB model to exhibit stabilized kinetic motifs, despite they both having 10 kinetic parameters). Note that our approach is quite general and can be integrated with other approaches. For instance, the Latin hypercube sampling (LHS) used here (and as in [33]) can be replaced by other methods, such as those mentioned above, to identify the kinetic solutions needed in the subsequent enrichment tests. Results from an experiment of combining LHS and genetic algorithm (GA) showed that 10^3^ of LHS sampling followed by 100 generations of GA optimization yielded a similar set of kinetic motifs for NFBLB (Additional file 1: Table S1), suggesting that optimization can help to find more solutions from a smaller initial parameter set, but in this case the hybrid approach did not reduce the computational cost (since it also needed a total of 10^5^ model evaluations), and could miss some of the marginally significant motifs (Additional file 1: Table S1). While further research is required to fully address the dimensionality problem of scaling up the system, complex biological networks are known to be composed of simple recurrent structural components [59-61]; a deeper understanding of these network components is an important first step toward a better understanding of the assemblage and functioning of a much larger system.

One interesting observation from the case studies of the NFBLB and the IFFLP (presented in Additional file 1: section VI) adaptation model is that the majority of parameters seem to contribute to only one single functionality (i.e. either sensitivity or precision, see Figure 3, Additional file 1: Figure S10). If the mathematical models are indeed mechanistic, this could have important biological implications as follows. The behavior or dynamics of a biological system is likely to be the result of intricate interactions of many biological processes. If a biological process has a major influence on all aspects of the systems behavior, then any changes to such a process may have a drastic impact on the system. Modularity is ubiquitous in biological systems as it provides an effective mechanism to corral damaging perturbations to local consequences [62]. Thus, to ensure system robustness, evolution might have favored a biological system with a fine division of labors (i.e. modularity) among different biological components or processes [63-65]. Here, in the kinetic-functionality association, we may have uncovered yet another example of nature’s modularity design manifestated in the organization of kinetic parameters.

Previous studies have shown that certain types and arrangements of network structures are required to produce certain types of system dynamics [33,66-68]; here, we have shown that, for a given network structure, certain types of values, or motifs, also exist for kinetic parameters in order to achieve specific system dynamics. Our results suggest that, as has been noted by others [69,70], system dynamics can put constraints on the values of kinetic parameters. The discovery of these motifs underscores the intricate inter-relationships between structure (i.e. topology) of the biological network, kinetic parameters of the reactions involved, and the function of the biological system. Delineation of these relationships by methods such as the one developed here, which is general and can be applied to other types of well defined dynamics, will greatly advance our understanding of the design principles of prototype biological systems.

## Conclusions

An increasing number of studies have revealed that complicated biological systems often share simple and universal design principles that are more understandable to biologists. The identification of motifs in biological networks is a prime example relating recurrent network structures to biological functions. Many studies have also argued for the importance of kinetic parameters in determining the dynamics of biological networks, but dissecting the association between system dynamics and kinetic parameters has been difficult. In this study, we have developed a methodology, akin to the enrichment analysis of gene expression profiles, to determine whether a preference of kinetic parameters adopting certain parameter values exists. Such preferences, or kinetic motifs, encapsulate the possible roles and functional constraints of kinetic parameters. Our analysis on models for the adaptation dynamics of the chemotaxis of *Escherichia coli* showed that design principles also exist from the perspective of kinetic parameters. Our methodology, owning to its generality and simplicity, provides a computational framework for understanding the kinetic mechanics of prototype biological networks.

## Competing interests

The authors declare that they have no competing interests.

## Authors’ contributions

Conceived and designed the experiments: AWTC MJH. Performed the experiments: AWTC. Analyzed the data: AWTC WCL PC MJH. Wrote the paper: AWTC WCL PC MJH. This work was supported by the Taiwan International Graduate Program and a grant from the National Science Council of Taiwan (NSC grant no. 100-2311-B-001-021) to MJH, and also by a visiting student fellowship to AWTC (NSC grant no. NSC98-2917-I-010-102). All authors read and approved the final manuscript.

## Supplementary Material

Additional file 1 **(I) Mechanisms of functional associations for the NFBLB model; (II) Additional analysis for the NFBLB model. Figure S1-S6**; (III) An analysis of computational cost; (IV) Simulation for a model of two linked NFBLPs (NFBLP^2^). **Figure S7**; (V) Results for GA-augmented simulations of the NFBLP model. **Table S1**; (VI) Results for the IFFLP model. **Figure S8-S11** and **Table S2-S3**. Click here for file
