# Supplementary material for: Understanding system dynamics of an adaptive enzyme network from globally profiled kinetic parameters
Source: BMC Syst Biol. 2014 Jan 15;8:4. doi: 10.1186/1752-0509-8-4 (PMC3896785; doi:10.1186/1752-0509-8-4)
Supplement: Additional file 1 — (I) Mechanisms of functional associations for the NFBLB model; (II) Additional analysis for the NFBLB model. Figure S1-S6; (III) An analysis of computational cost; (IV) Simulation for a model of two linked NFBLPs (NFBLP2). Figure S7; (V) Results for GA-augmented simulations of the NFBLP model. Table S1; (VI) Results for the IFFLP model. Figure S8-S11 and Table S2-S3. [file 1752-0509-8-4-S1.pdf]

Supplemental Data

I. Mechanisms of functional associations for the NFBLB model

An analytical analysis

Examining the rate equations (equation (7)) analytically via intuitive deductions can provide possible explanations for the observed association between kinetic motifs and perfect adaptation. Let's first focus on sensitivity which refers to the sharp increase in the concentration of activated node *C* immediately after the introduction of an external signal. For this to occur, the activation rate of node *C* must be high (the first term (v_{C1}) of the rate equation d*C*/dt in equation (7)), and this favors a small value for K_{AC}, consistent with its 1st value class motif (Table 1). Moreover, the NFBLB model assumes that the external signal activates node *C* indirectly via node *A*, which is negatively regulated by node *B*. Thus, a high deactivation rate for node *B* (the second term (v_{B2}) of the rate equation d*B*/dt in equation (7)) should result in a lower deactivation rate for node *A*, leading consequently to a rapid increase in the concentration of activated node *C*, and a small value (1st value class motif in Table 1) for K_{F_BB} is desirable for achieving this as it can result in a high deactivation rate for node *B*.

As for precision, it essentially refers to whether a system can return to its initial state after being perturbed by external signals. Dynamically speaking, the system is in a stable state, and any deviations from steady state must diminish in order for precision to occur. Thus, mechanisms that increase the deactivation rates of node *A* and node *C*, as well as those rendering a high persistent level of activated node *B* (note that node *B* is an inhibitor of node *A*) should result in high precision. From equation (7), a high deactivation rate for node *A* is possible with large values for k_{BA} and/or small values for K_{BA}, while node *C* can be deactivated at an elevated rate with

small values for K_{F_CC}. As for a high concentration of activated node *B*, this can be achieved with small values for K_{AB} and k_{F_BB}. Note that, as mentioned in the main text, if we take values within the first three value classes as small and within the last two as large, the relative magnitudes of these parameters as deduced from analytical analysis are in accord with their corresponding value class motifs obtained from the enrichment tests (Table 2), and also with those deduced from the experimental data for the *E. coli* system (Table 3). Our statistical tests also indicate an important role for K_{F_CC} in the sensitivity part, and although this was not obvious from the analytical deduction, it was suggested by a more rigorous analysis of simple enzymatic reactions similar to the rate equation of node *C* ([45], and equation (11) in the main text).

Analytical derivation of sensitivity indices

The NFBLB model in the vector form is:

$$\frac{ds}{dt} = f(s,p) = \begin{cases} f_1 = \dfrac{dA}{dt} = Ik_{IA}\dfrac{(1-A)}{(1-A)+K_{IA}} - Bk_{BA}\dfrac{A}{A+K_{BA}} \\ f_2 = \dfrac{dB}{dt} = Ak_{AB}\dfrac{(1-B)}{(1-B)+K_{AB}} - F_B k_{F_BB}\dfrac{B}{B+K_{F_BB}} \\ f_3 = \dfrac{dC}{dt} = Ak_{AC}\dfrac{(1-C)}{(1-C)+K_{AC}} - F_C k_{F_CC}\dfrac{C}{C+K_{F_CC}} \end{cases},$$

$$s = \{A, B, C\}, \qquad \text{(S1)}$$

$$p = \{k_{AB}, k_{AC}, k_{BA}, k_{F_BB}, k_{F_CC}, K_{AB}, K_{AC}, K_{BA}, K_{F_BB}, K_{F_CC}\},$$

where *s* is the concentration vector of *A*, *B* and *C*, f_1, f_2, and f_3 are the rate equation of *A*, *B*, and *C*, respectively, and *p* is the parameter of interest. At steady state, $f_1 = f_2 = f_3 = 0$, we can solve the sensitivity indices (change of concentration with respect to change of parameter) at steady state analytically as follows,

$$\frac{ds}{dp} = -\left[\frac{\partial f}{\partial s}\right]^{-1}\frac{\partial f}{\partial p} \qquad \text{(S2)}$$

where $\partial f/\partial s$ and $\partial f/\partial p$ are Jacobian matrices:

$$
\frac{\partial f}{\partial s}=\begin{bmatrix}
\frac{\partial f_1}{\partial A} & \frac{\partial f_1}{\partial B} & \frac{\partial f_1}{\partial C}\\
\frac{\partial f_2}{\partial A} & \frac{\partial f_2}{\partial B} & \frac{\partial f_2}{\partial C}\\
\frac{\partial f_3}{\partial A} & \frac{\partial f_3}{\partial B} & \frac{\partial f_3}{\partial C}
\end{bmatrix}
$$

$$
=\begin{bmatrix}
\frac{-Ik_{IA}}{(1-A)+K_{IA}}+\frac{I(1-A)k_{IA}}{[(1-A)+K_{IA}]^2}+\frac{-Bk_{BA}}{A+K_{BA}}+\frac{ABk_{BA}}{(A+K_{BA})^2} & \frac{-Ak_{BA}}{A+K_{BA}} & 0\\
\frac{k_{AB}(1-B)}{(1-B)+K_{AB}} & \frac{-Ak_{AB}}{(1-B)+K_{AB}}+\frac{A(1-B)k_{AB}}{[(1-B)+K_{AB}]^2}+\frac{-F_Bk_{F_BB}}{B+K_{F_BB}}+\frac{BF_Bk_{F_BB}}{(B+K_{F_BB})^2} & 0\\
\frac{k_{AC}(1-C)}{(1-C)+K_{AC}} & 0 & \frac{-Ak_{AC}}{(1-C)+K_{AC}}+\frac{A(1-C)k_{AC}}{[(1-C)+K_{AC}]^2}+\frac{-F_Ck_{F_CC}}{C+K_{F_CC}}+\frac{CF_Ck_{F_CC}}{(C+K_{F_CC})^2}
\end{bmatrix} \tag{S3}
$$

$$
\frac{\partial f}{\partial p}=\begin{bmatrix}
\frac{\partial f_1}{\partial k_{AB}} & \frac{\partial f_1}{\partial k_{AC}} & \frac{\partial f_1}{\partial k_{BA}} & \frac{\partial f_1}{\partial k_{F_BB}} & \frac{\partial f_1}{\partial k_{F_CC}} & \frac{\partial f_1}{\partial K_{AB}} & \frac{\partial f_1}{\partial K_{AC}} & \frac{\partial f_1}{\partial K_{BA}} & \frac{\partial f_1}{\partial K_{F_BB}} & \frac{\partial f_1}{\partial K_{F_CC}}\\
\frac{\partial f_2}{\partial k_{AB}} & \frac{\partial f_2}{\partial k_{AC}} & \frac{\partial f_2}{\partial k_{BA}} & \frac{\partial f_2}{\partial k_{F_BB}} & \frac{\partial f_2}{\partial k_{F_CC}} & \frac{\partial f_2}{\partial K_{AB}} & \frac{\partial f_2}{\partial K_{AC}} & \frac{\partial f_2}{\partial K_{BA}} & \frac{\partial f_2}{\partial K_{F_BB}} & \frac{\partial f_2}{\partial K_{F_CC}}\\
\frac{\partial f_3}{\partial k_{AB}} & \frac{\partial f_3}{\partial k_{AC}} & \frac{\partial f_3}{\partial k_{BA}} & \frac{\partial f_3}{\partial k_{F_BB}} & \frac{\partial f_3}{\partial k_{F_CC}} & \frac{\partial f_3}{\partial K_{AB}} & \frac{\partial f_3}{\partial K_{AC}} & \frac{\partial f_3}{\partial K_{BA}} & \frac{\partial f_3}{\partial K_{F_BB}} & \frac{\partial f_3}{\partial K_{F_CC}}
\end{bmatrix}
$$

$$
=\begin{bmatrix}
0 & 0 & \frac{-BA}{A+K_{BA}} & 0 & 0 & 0 & 0 & \frac{Bk_{BA}A}{(A+K_{BA})^2} & 0 & 0\\
\frac{A(1-B)}{(1-B)+K_{AB}} & 0 & 0 & \frac{-F_BB}{B+K_{F_BB}} & 0 & \frac{-Ak_{AB}(1-B)}{[(1-B)+K_{AB}]^2} & 0 & 0 & \frac{F_Bk_{F_BB}B}{(B+K_{F_BB})^2} & 0\\
0 & \frac{A(1-C)}{(1-C)+K_{AC}} & 0 & 0 & \frac{-F_CC}{C+K_{F_CC}} & 0 & \frac{-Ak_{AC}(1-C)}{[(1-C)+K_{AC}]^2} & 0 & 0 & \frac{F_Ck_{F_CC}C}{(C+K_{F_CC})^2}
\end{bmatrix} \tag{S4}
$$

II. Additional analysis for the NFBLB model

In this section, we computed the sensitivity indices of the NFBLB model using analytical derivation (equations S2-S4) and numerical estimations of AMIGO (Figure S1); compared the bootstrap results of AMIGO with our kinetic motifs (Figure S2); presented the results of additional simulations (Figure S3), of using different PR and SN thresholds (Figure S4) and of using additional value classes (Figure S5); determined the number of sampling/simulations required for converging kinetic motifs (Figure S6).

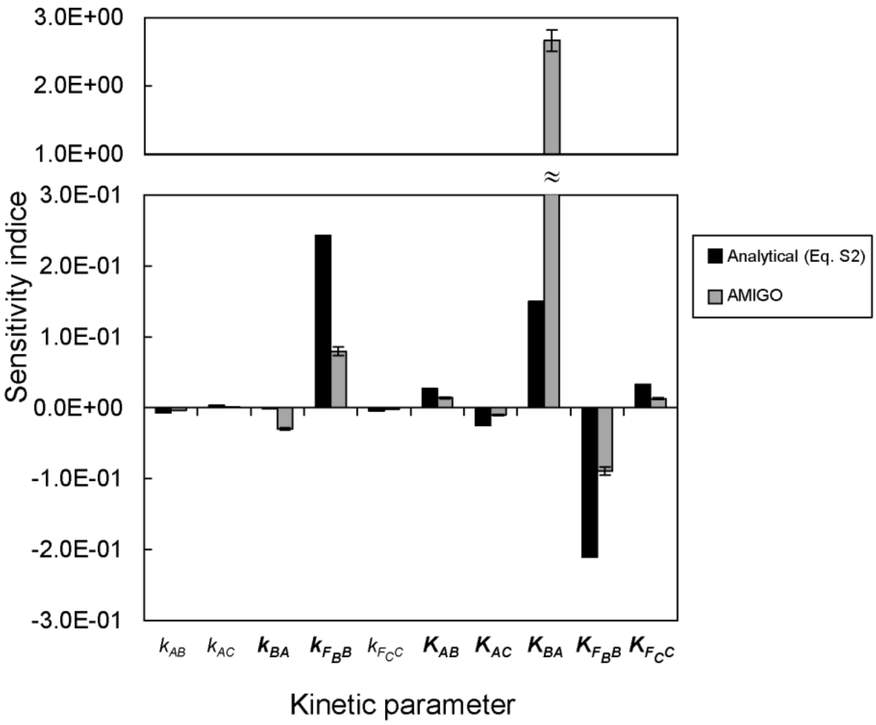

Figure S1 - Sensitivity indices from analytical derivation and AMIGO for the NFBLB model.

Shown are sensitivity indices (change of the concentration of node *C* with respect to change of the indicated kinetic parameter) obtained from analytical derivation (equation S2) and numerical estimation of AMIGO [56]. Data were computed using the parameter set of a specific solution chosen randomly from the 74 kinetic solutions of the NFBLB model. This solution was: (k_{BA}, k_{AB}, k_{FbB}, k_{AC}, k_{FcC}) = (7.52, 35, 0.5, 69.1, 56.83) and (K_{BA}, K_{AB}, K_{FbB}, K_{AC}, K_{FcC}) = (0.001, 0.002, 0.01, 0.005, 7.24); and, for analytical derivation (equation S2), the steady-state species concentration of ([*A*], [*B*], [*C*]) = (0.0131, 0.1051, 0.0236). Error bars denote the mean squared errors of AMIGO's sensitivity indices resulting from 2,400 evaluations at time points uniformly distributed over a period of 40 minutes. Note that the kinetic parameters in bold are those showing value class biases (i.e. having a motif, see Figure 3 in the main text) and they are also those having non-negligible sensitivities from the analytical derivation or AMIGO's numerical determination.

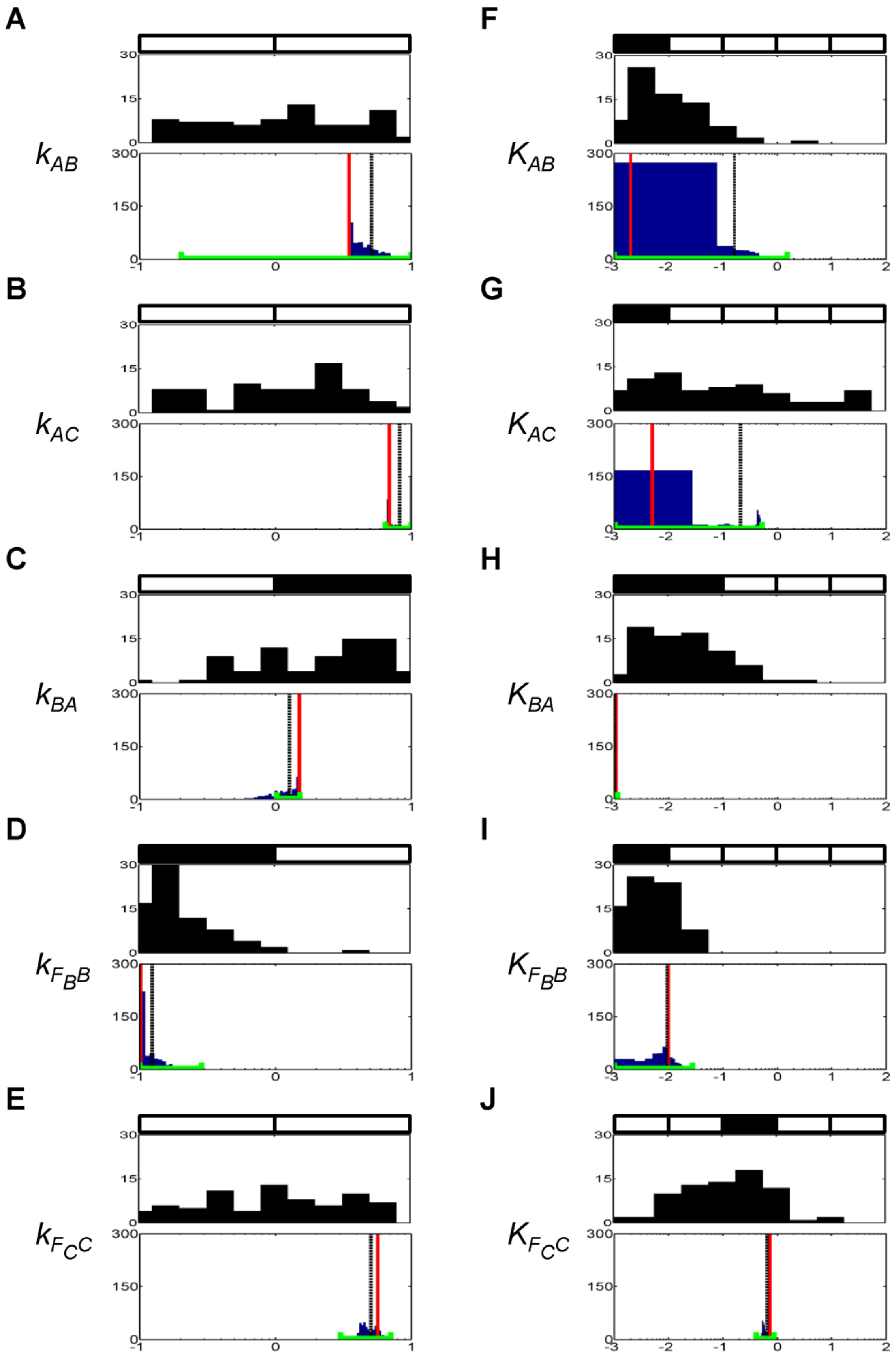
A
B
C
D
E
F
G
H
I
J
k_{AB}
k_{AC}
k_{BA}
k_{F_BB}
k_{F_CC}
K_{AB}
K_{AC}
K_{BA}
K_{F_BB}
K_{F_CC}

Figure S2 - Motifs and distributions of kinetic parameters in comparison with results of bootstrap analysis derived for one kinetic solution of the NFBLB model.

The motif (filled box in top panel) and distribution (middle panel) of each of the 10 kinetic parameters obtained from the 74 kinetic solutions of the NFBLB model are identical to those presented in Table 2 and Figure 3 in the main text. Bottom panels show the average (black vertical line), distribution histograms (in blue), and confidence intervals (bracketed in green) obtained from AMIGO [56] for 500 bootstrap replicates using the parameter set (red vertical line) of one of the 74 kinetic solutions as reference. Values of the parameters for this particular kinetic solution are provided in Figure S1. AMIGO's bootstrap analysis for this kinetic solution took 1hr and 20 min on a personal computer with an Intel quad-core CPU (2.66 GHz); in comparison, it took 2 hrs to identify/analyze the 74 kinetic solutions (from 10^5 samples) by our method on the same computer.

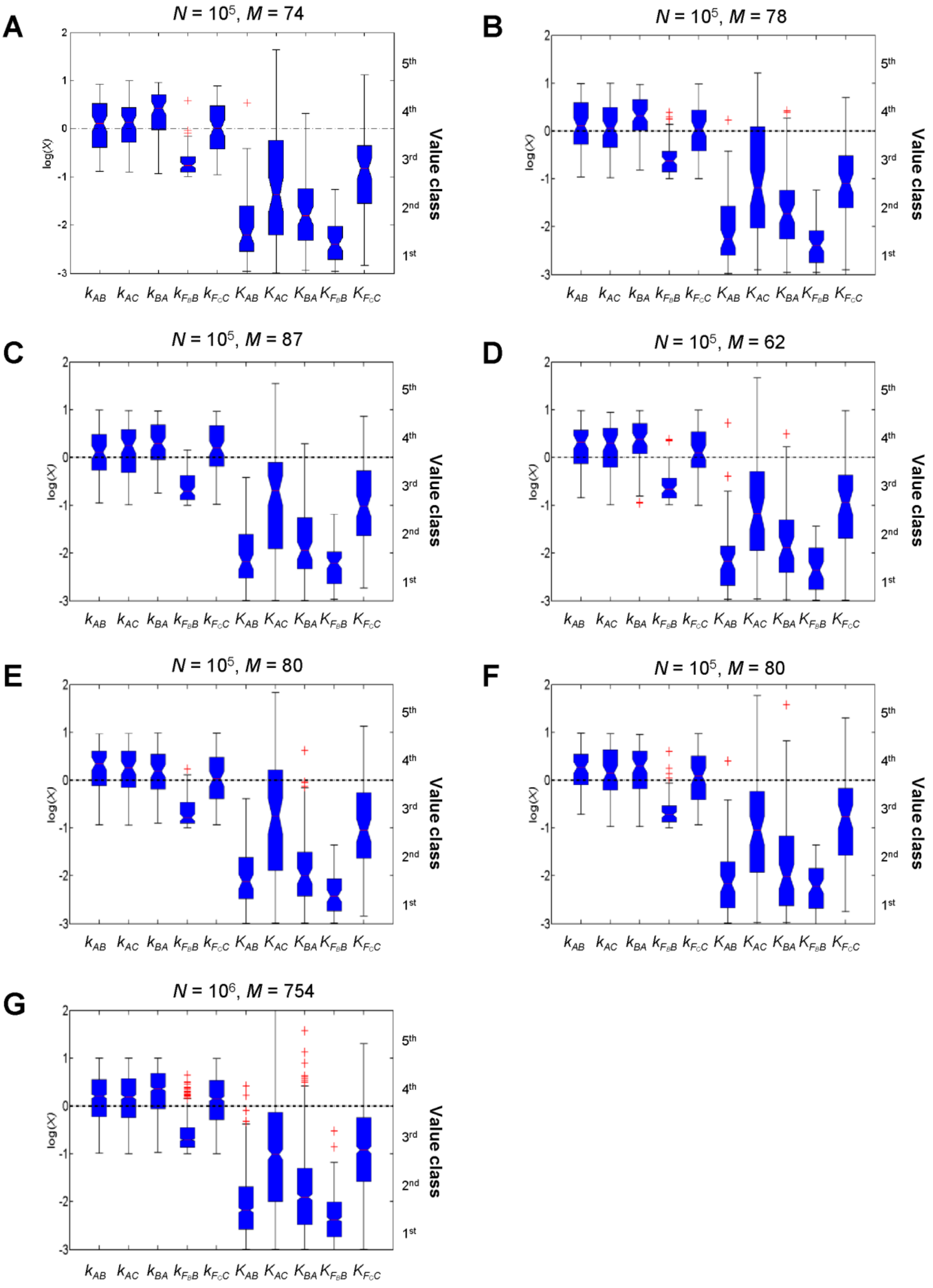
A
N = 10^5, M = 74
B
N = 10^5, M = 78
C
N = 10^5, M = 87
D
N = 10^5, M = 62
E
N = 10^5, M = 80
F
N = 10^5, M = 80
G
N = 10^6, M = 754
log(X)
Value class
1st
2nd
3rd
4th
5th
2
1
0
-1
-2
-3
k_AB k_AC k_BA k_FBB k_FCC K_AB K_AC K_BA K_FBB K_FCC

Figure S3 - Distributions of kinetic parameters from additional simulations of the NFBLB model.

A is identical to Figure 2 in the main text. B, C, D, E, F are results of five replicates each, like A, was carried out for a total of N=10^5 sampling/simulations. G: the results of 10^6 sampling/simulations. M is the number of kinetic solutions; all other notations are the same as those described in Figure 2 in the main text.

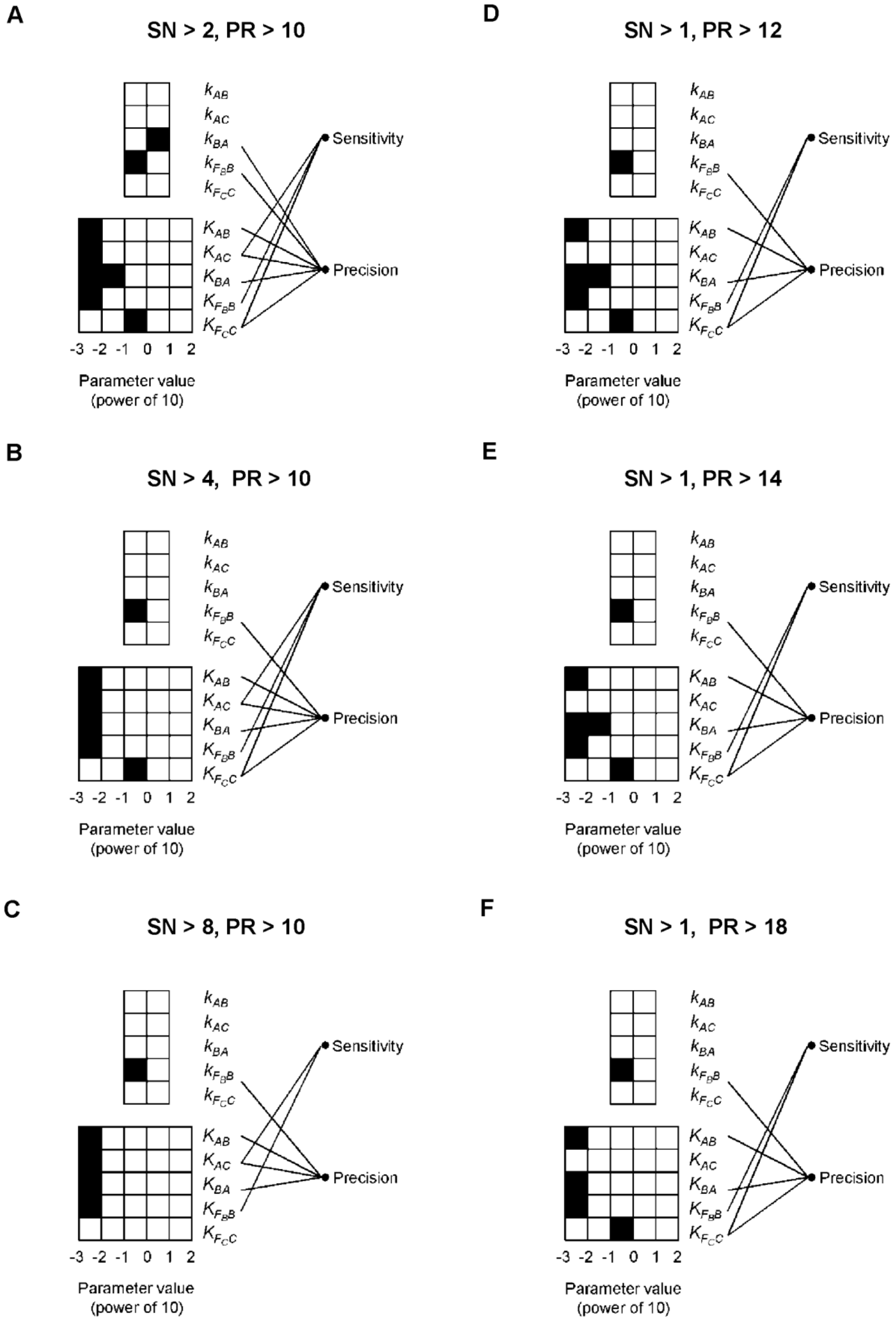
A
SN > 2, PR > 10
D
SN > 1, PR > 12
B
SN > 4, PR > 10
E
SN > 1, PR > 14
C
SN > 8, PR > 10
F
SN > 1, PR > 18
Sensitivity
Precision
Parameter value
(power of 10)

Figure S4 - Kinetic motifs and functionality networks using different thresholds of sensitivity (SN) and precision (PR) for the NFBLB model.

As the thresholds became more stringent, motifs may reduce their value range or even disappear (e.g. k_{BA}, K_{AC} in D, E; k_{BA}, K_{AC} and K_{BA} in F), but the main features of the motifs and the resulting functionality networks remained unchanged from those described in the main text for SN > 1 and PN > 10 (i.e, Figure 3). All the notations are the same as those described in Figure 3 in the main text.

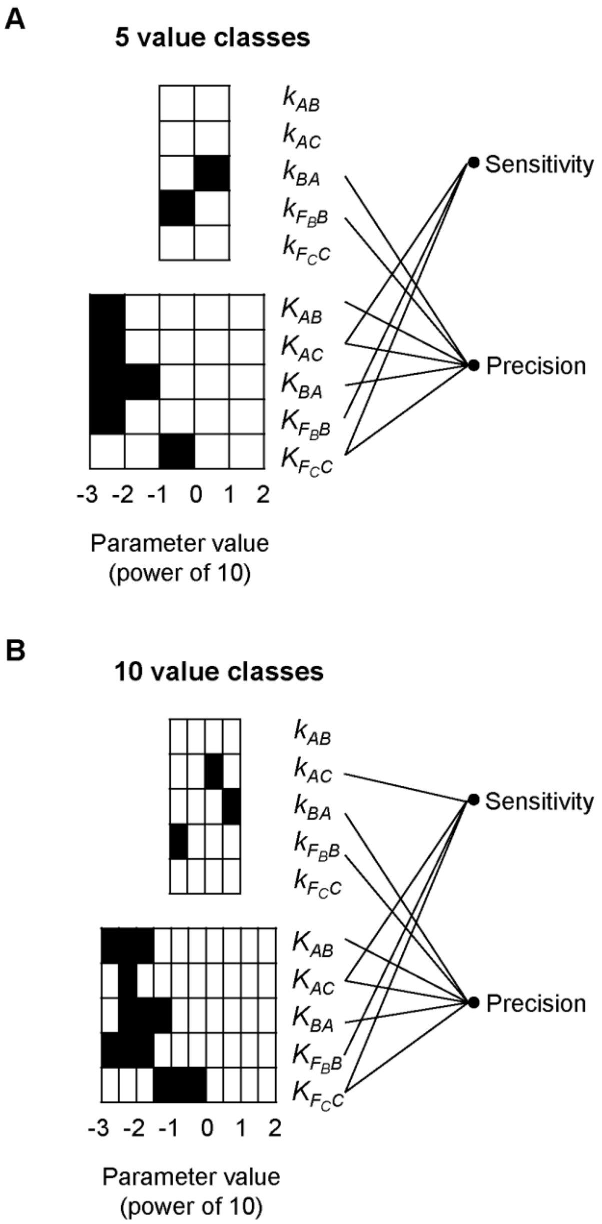

Figure S5 - Kinetic motifs using different number of value classes for the NFBLB model.

Doubling the number of value classes (reducing from 1 log-scale to 0.5 log-scale for each class) may change the value range of the parameter motif, and new motif with a reduced value range (e.g. k_{AC}) may also appear. However, the overall features of the motifs remained similar. The notations are the same as those described in Figure 3 in the main text.

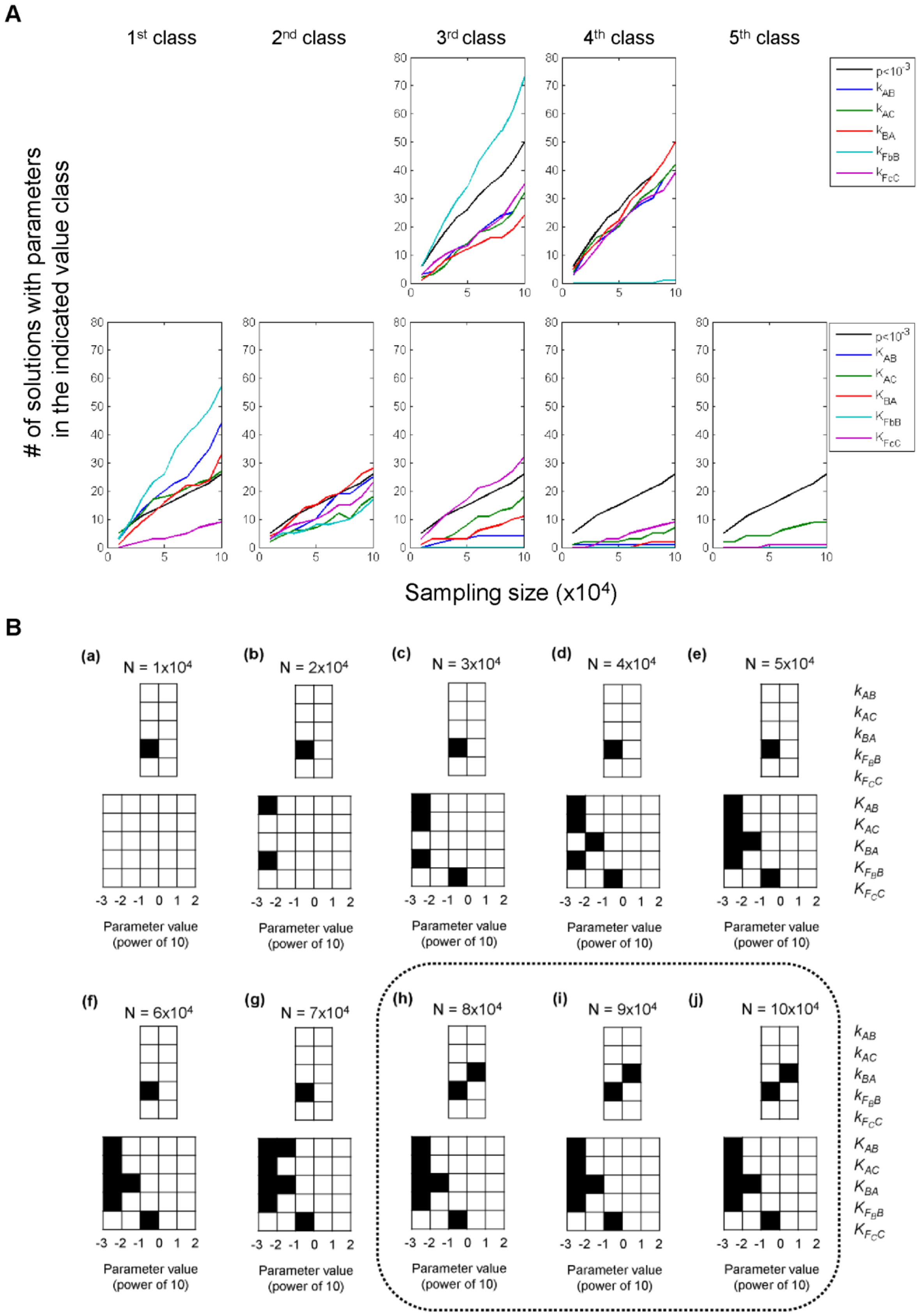
A
1st class
2nd class
3rd class
4th class
5th class
p<10-3
kAB
kAC
kBA
kFbB
kFcC
# of solutions with parameters in the indicated value class
Sampling size (x10^4)
B
(a) N = 1x10^4
(b) N = 2x10^4
(c) N = 3x10^4
(d) N = 4x10^4
(e) N = 5x10^4
(f) N = 6x10^4
(g) N = 7x10^4
(h) N = 8x10^4
(i) N = 9x10^4
(j) N = 10x10^4
kAB
kAC
kBA
kFBB
kFCC
KAB
KAC
KBA
KFBB
KFCC
Parameter value (power of 10)

Figure S6 - Number of kinetic solutions and motifs identified as a function of sampling size for the NFBLP model.

(A) Number of kinetic solutions having parameters in the indicated value class at the indicated sampling size (i.e. number of simulations). The black line denotes the minimal number of solutions required to find kinetic motifs at the specified level of statistical significance (p-value $\leq 10^{-3}$), as determined by the hypergeometric distribution (equation 10) using the empirical data of the simulation (color lines). Thus, when the number of solutions for a specific parameter (a specific color line) is greater than that of the black line, this parameter exhibits a motif with p-value $\leq 10^{-3}$. (B) The resulting kinetic motifs as a function of sampling size (number of simulations). The motifs exhibited no more changes in the last three sets of simulations (boxed by dash lines); in other words, the minimal number of sampling/simulations to obtain stabilized kinetic motifs was 8×10^4 for the NFBLP model. Note that these motifs are identical to those presented in Figure 3 in the main text.

III. An analysis of computational cost

The three steps of our method are: 1) sampling and simulation, 2) identification of kinetic motifs, and 3) functional association test. Let $\boldsymbol{N}$ be the sample size, $\boldsymbol{k}$ the number of parameters, $\boldsymbol{v}$ the number of value classes, $\boldsymbol{m}$ the number of kinetic motifs, and $\boldsymbol{f}$ the number of functional elements to be associated with parameters (e.g. 2, sensitivity (SN) and precision (PR) of adaptation, in this study). Our computation cost can be estimated as follows.

1) For the sampling/simulation step, the computation cost is $\boldsymbol{N}$ model evaluations, i.e. $\boldsymbol{N}$ simulations for a total of $\boldsymbol{N}$ simulated samples ($\boldsymbol{N}$ sets of parameters).
2) For the identification of kinetic motifs step, the computation cost is $\boldsymbol{kv}$ evaluations of the enrichment test (equation (10) in the main text), because each kinetic parameter is tested for each of the $\boldsymbol{v}$ value classes.
3) Upon identifying $\boldsymbol{m}$ kinetic motifs, each motif is tested by the Mann-Whitney *U*-test (see Method in the main text) for association with each of the $\boldsymbol{f}$ functional elements in both the motif and non-motif group. The computation cost for this step is estimated to be $\boldsymbol{3mf}$ evaluations (for each motif, 2 calculations of group means for each functional element plus 1 calculation of the *U*-test).

The total computation cost of the three sequential steps is therefore $\boldsymbol{N+kv+3mf}$ evaluations. Since $\boldsymbol{m} \le \boldsymbol{k}$, by replacing $\boldsymbol{m}$ with $\boldsymbol{k}$, the maximum computation cost is $\boldsymbol{N+k(v+3f)}$ evaluations. Because $\boldsymbol{N} >> \boldsymbol{k}$, $\boldsymbol{v}$ and $\boldsymbol{f}$, the computational complexity of our method is essentially equivalent to the order of, and therefore dominated by, $\boldsymbol{N}$.

IV. Simulation for a model of two linked NFBLPs (NFBLP^2)

To investigate the computational cost of our method for a larger network, the following mock model was simulated where two NFBLB modules were linked such that the output of module 1 was the input to module 2. This resulted in a network with twice the number of kinetic parameters to be sampled than the single NFBLB model studied in the main text. For simplicity, the same ‘perfect adaptation’ and all its thresholds of parameters as defined in the main text were followed to find kinetic solutions and, in the subsequent statistical analysis, kinetic motifs.

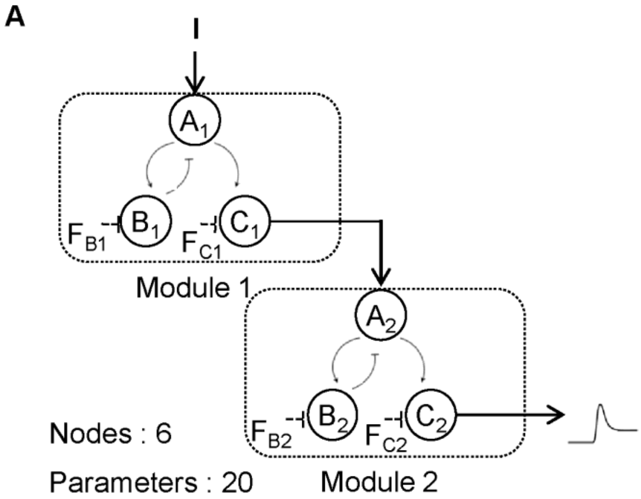

B

Module 1

$$\begin{cases} \dfrac{dA_1}{dt} = Ik_{IA_1}\dfrac{(1-A_1)}{(1-A_1)+K_{IA_1}} - B_1k_{B_1A_1}\dfrac{A_1}{A_1+K_{B_1A_1}} \\ \dfrac{dB_1}{dt} = A_1k_{A_1B_1}\dfrac{(1-B_1)}{(1-B_1)+K_{A_1B_1}} - F_{B_1}k_{F_{B1}B_1}\dfrac{B_1}{B_1+K_{F_{B1}B_1}} \\ \dfrac{dC_1}{dt} = A_1k_{A_1C_1}\dfrac{(1-C_1)}{(1-C_1)+K_{A_1C_1}} - F_{C_1}k_{F_{C1}C_1}\dfrac{C_1}{C_1+K_{F_{C1}C_1}} \end{cases}$$

Module 2

$$\begin{cases} \dfrac{dA_2}{dt} = C_1k_{C_1A_2}\dfrac{(1-A_2)}{(1-A_2)+K_{C_1A_2}} - B_2k_{B_2A_2}\dfrac{A_2}{A_2+K_{B_2A_2}} \\ \dfrac{dB_2}{dt} = A_2k_{A_2B_2}\dfrac{(1-B_2)}{(1-B_2)+K_{A_2B_2}} - F_{B_2}k_{F_{B2}B_2}\dfrac{B_2}{B_2+K_{F_{B2}B_2}} \\ \dfrac{dC_2}{dt} = A_2k_{A_2C_2}\dfrac{(1-C_2)}{(1-C_2)+K_{A_2C_2}} - F_{C_2}k_{F_{C2}C_2}\dfrac{C_2}{C_2+K_{F_{C2}C_2}} \end{cases}$$

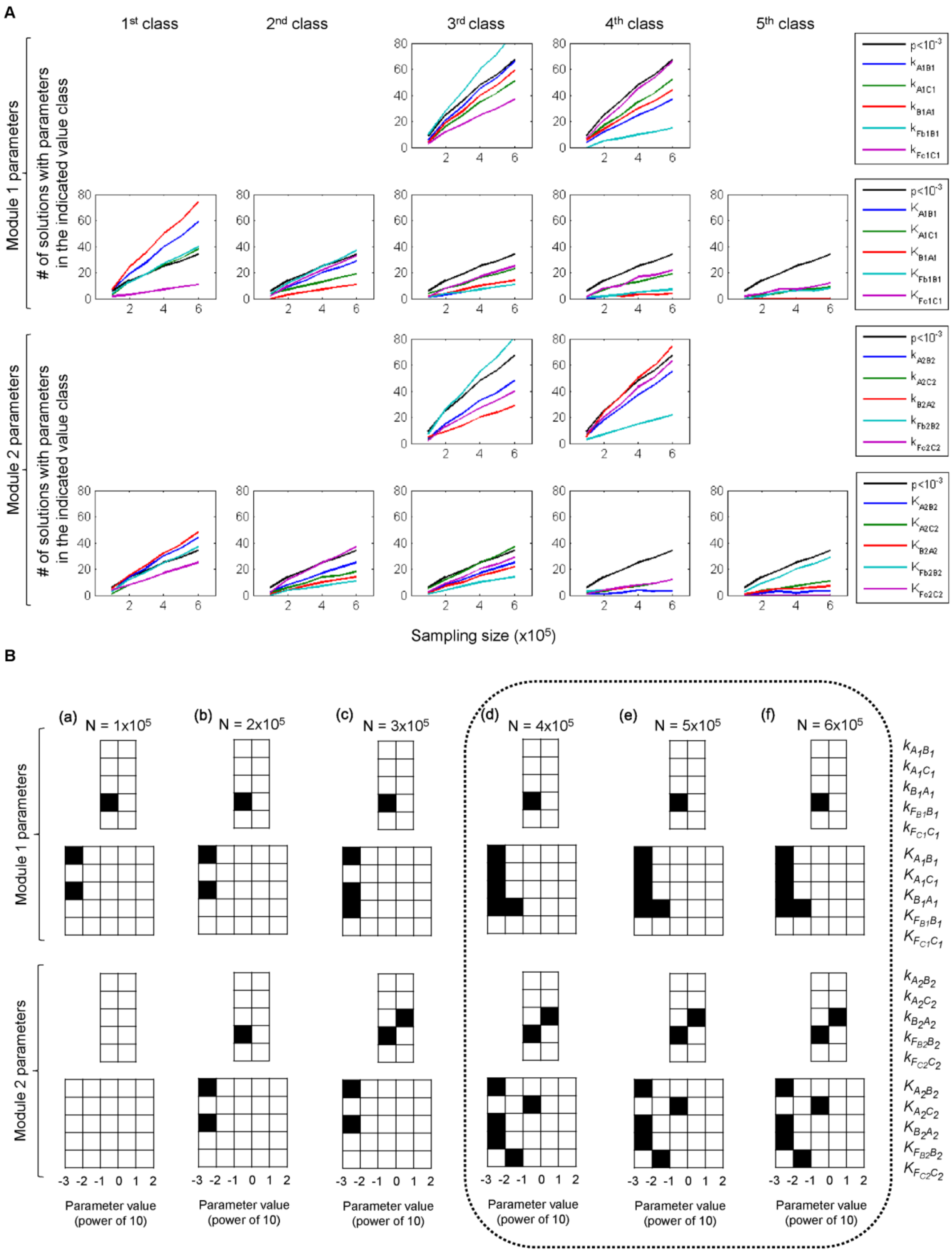
A
1st class
2nd class
3rd class
4th class
5th class
Module 1 parameters
Module 2 parameters
# of solutions with parameters in the indicated value class
Sampling size (x10^5)
B
(a) N = 1x10^5
(b) N = 2x10^5
(c) N = 3x10^5
(d) N = 4x10^5
(e) N = 5x10^5
(f) N = 6x10^5
Parameter value (power of 10)

Figure S7 - Number of kinetic solutions and motifs identified as a function of sampling size for the NFBLP^2 model.

(A) Number of kinetic solutions having parameters in the indicated value class at the indicated sampling size (i.e. number of simulations). The black line denotes the minimal number of solutions required to find kinetic motifs at the specified level of statistical significance (p-value ≤ 10^{-3}), as determined by the hypergeometric distribution (equation 10) using the empirical data of the simulation (color lines). Thus, when the number of solutions for a specific parameter (a specific color line) is greater than that of the black line, this parameter exhibits a motif with p-value ≤ 10^{-3}. (B) The resulting kinetic motifs as a function of sampling size (number of simulations). The motifs exhibited no more changes in the last three sets of simulations (boxed by dash lines); in other words, the minimal number of sampling/simulations to obtain stabilized kinetic motifs was $4x10^5$ for the NFBLP^2 model. Note that these motifs showed considerable similarity but were not identical to those of the NFBLP model because the input to the second NFBLP module of the NFBLP^2 network was different from that to the first (or single) NFBLP module.

V. Results for GA-augmented simulations of the NFBLP model.

In this section, we present simulation results from a LHS-GA hybrid approach, in which 1000 parameter sets sampled by the LHS method [35] were subsequently augmented by 100 generations of GA optimization. A transformed SN+PR score used in the computer program of Ma et al. [33] for adaptation dynamics was used as the objective function for the GA optimization. A subroutine from GAlib (a genetic algorithm library; http://lancet.mit.edu/ga/) was implemented for this hybrid approach. For each GA generation, the best 1000 individuals (parameter sets) were selected to create a new generation by operations of genetic crossover (at 0.9 rate) and mutation (at 0.01 rate). This GA cycle was repeated until 100 generations were reached, at which point the maximal score of the objective function had reached a plateau, indicative of convergence (see below). The results (of the kinetic motifs) obtained from three independent runs of this hybrid approach are presented in Table S1.

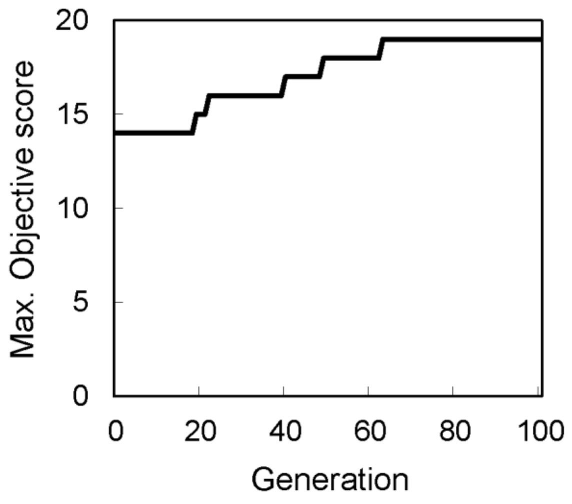

Table S1 - Results of parameter enrichment test for the NFBLB model from GA-augmented simulations.

GA run (Solutions)	Parameter	1st class [10^{-3},10^{-2}][a]			2nd class [10^{-2},10^{-1}]			3rd class [10^{-1},10^{0}]			4th class [10^{0},10^{1}]			5th class [10^{1},10^{2}]		
		x^b	y^c	p^d	x	y	p	x	y	p	x	y	p	x	y	p
**Run 1 (67)**	k_{AB}							13	29,737	9.6E-01	54	70,263	2.0E-02			
	k_{AC}							14	25,798	7.8E-01	53	74,202	1.4E-01			
	k_{BA}							10	21,625	8.8E-01	57	78,375	6.4E-02			
	k_{FBB}							66	87,984	**1.9E-04**	1	12,016	1.0E+00			
	k_{FCC}							5	18,465	9.9E-01	62	81,535	3.1E-03			
	K_{AB}	62	69,908	**1.2E-06**	4	11,772	9.1E-01	1	8,692	9.8E-01	0	5,215	9.7E-01	0	4,413	9.5E-01
	K_{AC}	17	23,124	2.7E-01	22	29,336	2.2E-01	21	34,543	6.6E-01	5	7,859	4.3E-01	2	5,138	6.8E-01
	K_{BA}	62	78,594	**5.2E-04**	5	11,477	8.0E-01	0	4,760	9.6E-01	0	3,051	8.7E-01	0	2,118	7.6E-01
	K_{FBB}	66	73,483	**1.2E-09**	1	5,541	8.9E-01	0	3,694	9.2E-01	0	8,196	1.0E+00	0	9,086	1.0E+00
	K_{FCC}	1	2,827	5.7E-01	6	9,138	4.1E-01	55	68,120	3.3E-03	5	14,076	9.2E-01	0	5,839	9.8E-01
**Run 2 (65)**	k_{AB}							17	40,220	9.9E-01	48	59,780	6.2E-03			
	k_{AC}							9	16,533	6.5E-01	56	83,467	2.3E-01			
	k_{BA}							4	14,303	9.6E-01	61	85,697	1.2E-02			
	k_{FBB}							65	91,424	**<2.2E-16**	0	8,576	1.0E+00			
	k_{FCC}							5	23,488	1.0E+00	60	76,512	**2.1E-04**			
	K_{AB}	62	68,942	**1.4E-08**	3	13,830	9.8E-01	0	6,865	9.9E-01	0	5,339	9.7E-01	0	5,024	9.6E-01
	K_{AC}	4	9,306	7.3E-01	21	30,241	3.0E-01	37	42,856	8.1E-03	2	12,421	9.9E-01	1	5,176	8.6E-01
	K_{BA}	62	77,683	**1.4E-05**	2	8,900	9.4E-01	1	8,498	9.8E-01	0	2,640	8.2E-01	0	2,279	7.8E-01
	K_{FBB}	63	70,789	**5.1E-09**	2	7,716	8.9E-01	0	4,543	9.5E-01	0	9,689	1.0E+00	0	7,263	9.9E-01
	K_{FCC}	0	2,974	8.6E-01	12	16,891	3.0E-01	48	64,450	4.1E-02	5	11,732	7.9E-01	0	3,953	9.3E-01
**Run 3 (66)**	k_{AB}							19	37,845	9.2E-01	47	62,155	4.8E-02			
	k_{AC}							7	15,354	8.1E-01	59	84,646	1.0E-01			
	k_{BA}							2	14,172	1.0E+00	64	85,828	**4.9E-04**			
	k_{FBB}							66	90,287	**<2.2E-16**	0	9,713	1.0E+00			
	k_{FCC}							8	20,223	9.4E-01	58	79,777	3.0E-02			
	K_{AB}	65	74,346	**3.1E-09**	0	8,842	1.0E+00	1	7,340	9.6E-01	0	5,661	9.8E-01	0	3,811	9.2E-01
	K_{AC}	13	13,812	6.5E-02	16	18,610	9.4E-02	28	45,385	6.4E-01	9	18,835	8.2E-01	0	3,358	9.0E-01
	K_{BA}	62	77,497	**6.5E-05**	4	10,428	8.3E-01	0	7,366	9.9E-01	0	2,723	8.4E-01	0	1,986	7.3E-01
	K_{FBB}	65	76,699	**2.5E-08**	1	7,074	9.5E-01	0	3,549	9.1E-01	0	6,610	9.9E-01	0	6,068	9.8E-01
	K_{FCC}	1	3,426	6.7E-01	36	40,424	7.3E-03	27	38,543	3.0E-01	2	10,066	9.7E-01	0	7,541	9.9E-01

[a] In square brackets are the intervals of parameter values for the indicated class.

[b] Out of the kinetic solutions, x is the number of solutions with the value of the indicated parameter belonging to the indicated value class.

[c] Out of a total of N ($=10^5$) parameter sets sampled (100 generations upon each of 1,000 initial parameter sets), y is the number of sets with the value of the indicated parameter belonging to the indicated value class.

[d] An enrichment test is considered statistically significant if its p-value$<10^{-3}$ and is highlighted in boldface. Based on *p*-values (using 10^{-3} as threshold), an enrichment state, i.e. motif, was assigned. Shaded p-values are those determined as a motif in Table 1 but not here in the GA-augmented hybrid method; note that these are the motifs exhibiting marginal significance statistically in Table 1 in the main text. The boxed p-value is the only new one found to be statistically significant (for Run 2 only) in the GA-augmented simulations.

VI. Results for the IFFLP model

The mathematical equations for the IFFLP model (Figure S8) are:

$$
\begin{aligned}
\frac{dA}{dt} &= \underbrace{Ik_{IA}\frac{(1-A)}{(1-A)+K_{IA}}}_{v_{A1}} - \underbrace{Bk_{BA}\frac{A}{A+K_{BA}}}_{v_{A2}} \\
\frac{dB}{dt} &= \underbrace{Ak_{AB}\frac{(1-B)}{(1-B)+K_{AB}}}_{v_{B1}} - \underbrace{Ck_{CB}\frac{B}{B+K_{CB}}}_{v_{B2}} \\
\frac{dC}{dt} &= \underbrace{Bk_{BC}\frac{(1-C)}{(1-C)+K_{BC}}}_{v_{C1}} - \underbrace{Ak_{AC}\frac{C}{C+K_{AC}}}_{v_{C2}}
\end{aligned}
\qquad \text{(S5)}
$$

where all the notations are the same as in equation (7) in the main text. For the IFFLP model, 10^4 sets of parameter values were sampled and 6,073 of them were found to exhibit adaptation dynamics, of which 131 satisfied the criteria for perfect adaptation (see Methods). The same procedures of analysis and statistical tests as described in the main text were followed, which produced a plot of kinetic parameter distributions (Figure S9), a kinetic-functionality network (Figure S10), a plot of correlation between kinetic parameters (Figure S11), and results from the enrichment (Table S2) and association (Table S3) statistical tests. These results indicated that catalytic rate constant k_{AB} was biased towards the 3rd values class, while k_{AC} and k_{BA} were both biased towards the 4th values class. The Michaelis-Menten constants K_{AC} and K_{BA} were biased towards the first two values classes, and K_{AB} and K_{CB} were biased towards one or both of the last two values classes. The other parameters, k_{BC}, k_{CB} and K_{BC}, did not show any biases. In addition, the four kinetic parameters, k_{AC}, k_{BA}, K_{AC} and K_{BA}, were shown to be highly significant in improving the sensitivity scores, while k_{AB}, K_{AB} and K_{BC} were significant in improving the precision scores. Again, like

in the NFBLB model, a kinetic-functionality network highlighting the relationship between kinetic parameters and functionalities of dynamics can be derived (Figure S8).

Below, we provide possible explanations for the observed associations between kinetic motifs and perfect adaptation for the IFFLP model. Here, the introduction of an external signal decreases the concentration of activated node *C* by the negative regulating effect from node *A* (Figure S8). For this to occur, the deactivation rate of node *C* must be high (the second term (v_{C2}) of the rate equation d*C*/dt from equation (S5)), and this can be achieved with a large value for k_{AC}, consistent with its 4th value class motif (Table S2). According to Goldbeter and Koshland [45], in order to ensure ultra-sensitivity, the system must be in the condition of saturation, which is in accord with our finding that both parameters K_{AC} and K_{BA} must take on small values (i.e. $< 10^0$, or value class 1st, 2nd, or 3rd).

From the topology of the network (Figure S8), one can also observe that node *A* decreases directly the concentration of node *C*, and after some delay it also increases the concentration of node *C* via node *B*. The latter route thus serves as means for returning node *C* to its pre-stimulated level, and to do so the inhibition mechanism of node *B* via node *C* must be kept at a slow rate, which justifies our numerical finding that K_{CB} takes on large values (4th and 5th value class) in order for the system to attain precision (as K_{CB} appears in the denominator of the inhibition rate of node *B*). Note that, like in the NFBLB model, we took values within the first three value classes as small and within the last two as large, and the relative magnitudes of these parameters as deduced from analytical analysis are in accord with their corresponding value class motifs obtained from the enrichment tests (Table S2).

However, like in the NFBLB model, our numerical results do not completely agree with all our intuitive explanations. For example, k_{BA} was found to be biased towards large values (4th value class), which can result in a high deactivation rate for node A and should damp the effect of input signal on node C. From this, we speculate that a large k_{BA} should cause high precision and should have no effect on the sensitivity part of the systems dynamics, but our numerical analysis showed the contrary (Table S3). Furthermore, according to our analytical analysis, K_{AB} should take on small values as this would result in a saturated region for the first term (v_{B1}) of the rate equation dB/dt in equation (S5), but our numerical results showed that it was in fact enriched in large values (Table S2). A close inspection of the kinetic solutions showed that a sizable fraction (47/131, or 36%; Table S2) of K_{AB} have small values (1st, 2nd and 3rd value class) despite it being shown to be statistically enriched in large values to produce perfect adaptation. This may imply a less stringent constraint on K_{AB} than on some of the other parameters, and the observed motif of K_{AB} represents a sufficient condition but not a necessary condition for producing adaptation dynamics. Here, we should note that the intuitive deductions do not provide necessary proofs of the numerical simulation results. More sophisticated mathematical treatments or experimental investigations are required to resolve any discrepancies.

Finally, like in the NFBLB model, we checked whether kinetic parameters work in a cooperative way with each other when contributing to the system's adaptation dynamics. Figure S11 shows the results of correlation tests performed on all pairs of the seven kinetic parameters exhibiting value class biases. We identified two significant positive correlations (*p-value* < 0.05) in (k_{AB}, K_{AB}) and (K_{AC}, K_{CB}) pairs, and three significant negative correlations in (k_{AC}, K_{AB}), (k_{AB}, K_{AC}) and (k_{AB}, K_{CB}) pairs.

Again, like in the NFBLB model, with the exceptions of k_{AC} and K_{AC}, all the correlated parameters contributed to system's precision (Figure S10), suggesting that they work in a cooperative manner in the precision mechanism of adaptation dynamics. In contrast, kinetic parameters contributing to the system's sensitivity (i.e. k_{AC}, k_{BA}, K_{AC} and K_{BA}, see Figure S10) were not correlated with each other, implying that they function independently in the sensitivity mechanism.

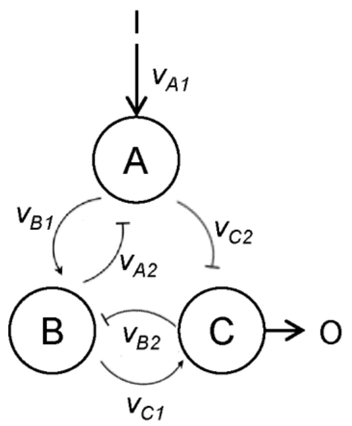

Figure S8. The incoherent feed-forward loop model for adaptive enzyme dynamics.

An enzyme network with an incoherent feed-forward loop, known as the IFFLP model [33], where v_{n1} (v_{n2}) (n = A, B or C) represents the activation (deactivation) process of the rate equation for node n.

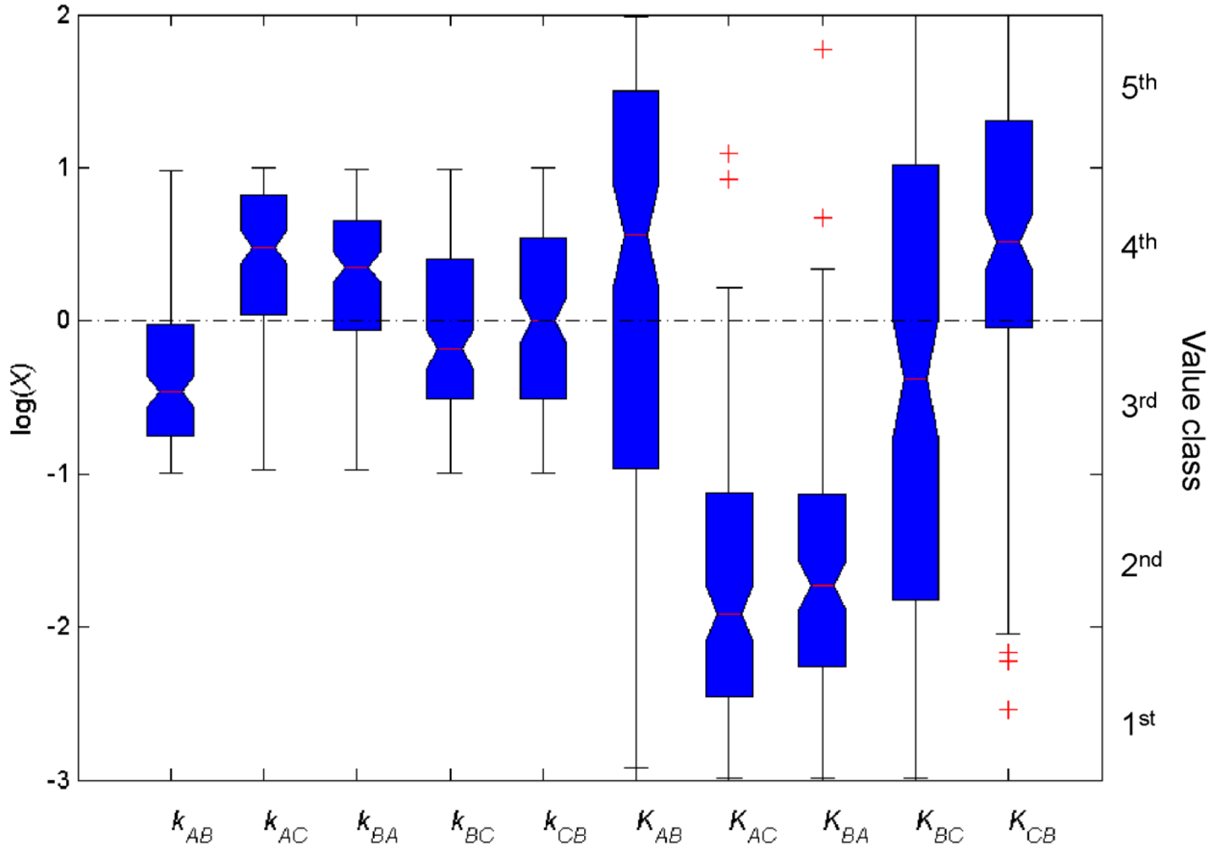

Figure S9 - Distributions of kinetic parameters for the IFFLP model.
Distributions of parameter values obtained from a total of 131 kinetic solutions exhibiting perfect adaptation for the IFFLP model. Each tick on the x axis is a specific catalytic rate constant *k* or Michaelis-Menten constant *K*, and the values of the parameters are in power of 10, which are divided into five value classes as indicated at the right of the figure. On each data box, the contracted center is the median, while the edges of the box are the 25th and 75th percentiles of the distribution.

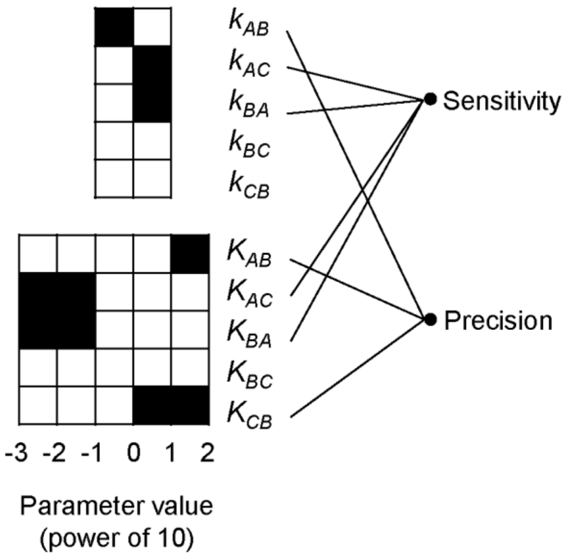

Figure S10 - A kinetic functionality network for the IFFLP model.
A bipartite network connecting kinetic parameters to functionalities (sensitivity and precision) of the adaptation dynamics. On the left are kinetic motifs emerged from the enrichment tests (see Methods), where filled boxes represent enriched values bounded by the indicated power of 10 for the indicated parameter. On the right are different functionalities (sensitivity and precision) of adaptation dynamics. A connection between a kinetic parameter and functionality was established if the association between the two was determined to be significant in the statistical test (see Methods).

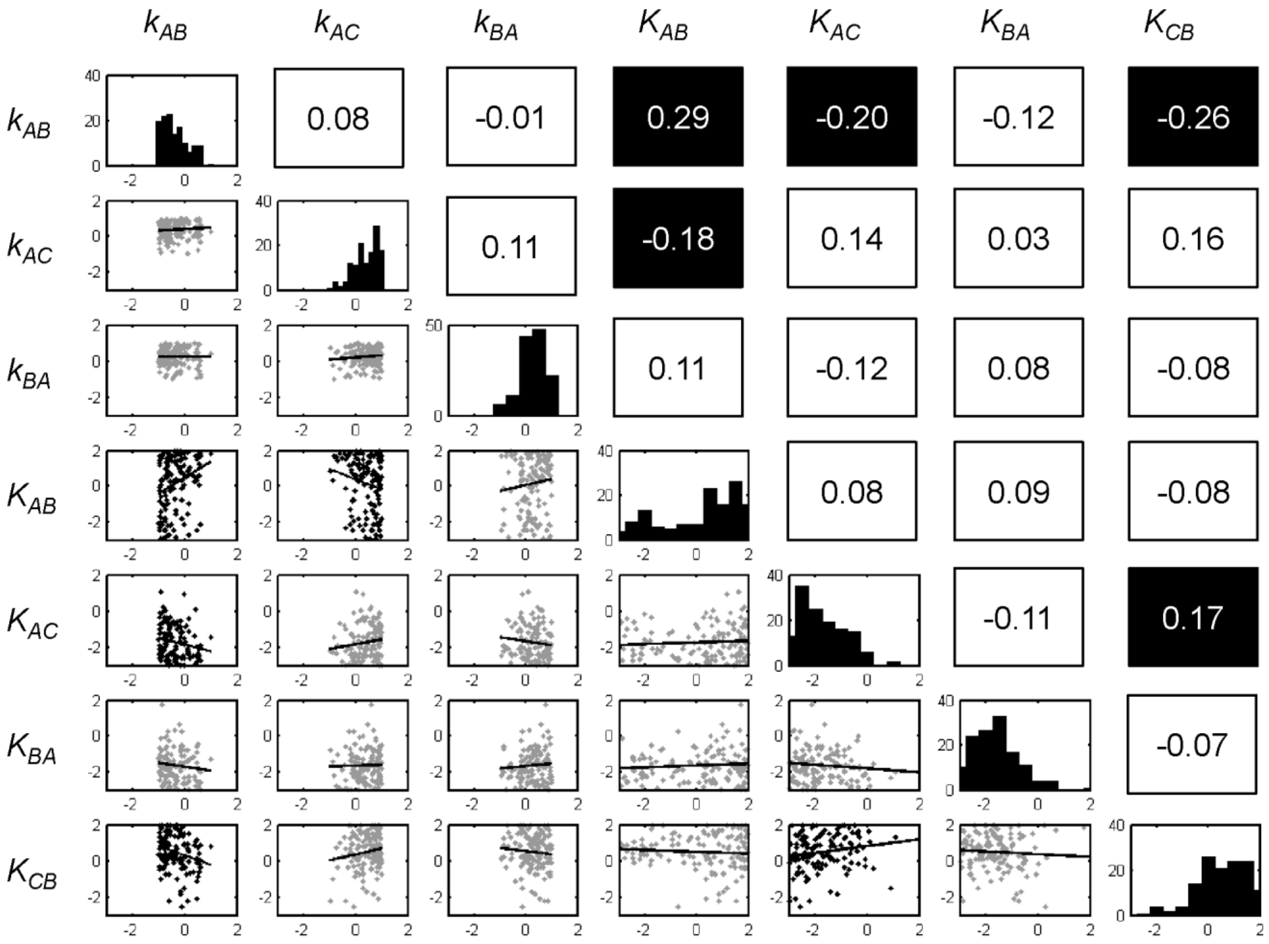

Figure S11 - Correlation between kinetic parameters for the IFFLP model.

The Pearson correlation coefficients between pairs of the seven parameters that exhibited value class enrichment are shown in the top-right triangle, where a box is colored black if the corresponding correlation is significant (p-value < 0.05). In the bottom left triangle, the scatter plots of the paired parameters are shown. On the diagonal are occurrence distributions of individual kinetic parameters.

Table S2 - Results of parameter enrichment test for the IFFLP model.

Parameter	1st class $[10^{-3},10^{-2}]$[a]			2nd class $[10^{-2},10^{-1}]$			3rd class $[10^{-1},10^{0}]$			4th class $[10^{0},10^{1}]$			5th class $[10^{1},10^{2}]$		
	x[b]	*y*[c]	*p*[d]	*x*	*y*	*p*	*x*	*y*	*p*	*x*	*y*	*p*	*x*	*y*	*p*
k_{AB}							102	4,975	**1.2E-11**	29	5,025	1.0E+00			
k_{AC}							30	4,999	1.0E+00	101	5,001	**5.2E-11**			
k_{BA}							36	5,050	1.0E+00	95	4,950	**2.1E-08**			
k_{BC}							79	4,947	4.8E-03	52	5,053	9.9E-01			
k_{CB}							66	4,990	4.2E-01	65	5,010	5.1E-01			
K_{AB}	20	1,943	8.7E-01	12	1,960	1.0E+00	15	2,035	9.9E-01	34	2,029	4.5E-02	50	2,033	**6.8E-07**
K_{AC}	60	1,978	**2.0E-11**	40	1,959	**9.0E-04**	26	2,048	5.2E-01	4	1,946	1.0E+00	1	2,069	1.0E+00
K_{BA}	44	2,006	**8.4E-05**	61	2,051	**1.6E-11**	20	2,016	9.1E-01	5	1,941	1.0E+00	1	1,986	1.0E+00
K_{BC}	27	1,986	3.6E-01	22	1,999	7.9E-01	24	2,045	6.9E-01	25	2,037	5.9E-01	33	1,933	3.8E-02
K_{CB}	4	1,998	1.0E+00	5	2,061	1.0E+00	25	1,943	4.9E-01	56	1,983	**4.9E-10**	41	2,015	**8.3E-04**

e. In square brackets are the intervals of parameter values for the indicated class.

f. Out of *M* (=131) kinetic solutions, x is the number of solutions with the value of the indicated parameter belonging to the indicated value class.

g. Out of a total of *N* (=10^4) parameter sets sampled, y is the number of sets with the value of the indicated parameter belonging to the indicated value class.

h. An enrichment test is considered statistically significant if its p-value<10^{-3} and is highlighted. Based on *p*-values (using 10^{-3} as threshold), an enrichment state, i.e. motif, was assigned.

Table S3 - Functional association test results for the IFFLP model.

Para-meter	Motif [m][a]	Non-motif [~m]	Precision Test[b]			Sensitivity Test[b]			Func-tion[c]
			Pr_m	$Pr_{\sim m}$	z	Sn_m	$Sn_{\sim m}$	z	
k_{AB}	$[10^{-1},10^{0}]$	$[10^{0},10^{1}]$	2.04	1.89	**4.44**	-1.65	-1.70	2.20	PR
k_{AC}	$[10^{0},10^{1}]$	$[10^{-1},10^{0}]$	1.72	2.22	-12.73	-1.34	-2.02	**17.64**	SN
k_{BA}	$[10^{0},10^{1}]$	$[10^{-1},10^{0}]$	1.88	2.08	-5.17	-1.51	-1.88	**9.58**	SN
K_{AB}	$[10^{1},10^{2}]$	$[10^{-3},10^{1}]$	2.06	1.94	**3.74**	-1.70	-1.67	0.14	PR
K_{AC}	$[10^{-3},10^{-1}]$	$[10^{-1},10^{2}]$	1.55	2.22	-18.60	-1.13	-2.01	**24.19**	SN
K_{BA}	$[10^{-3},10^{-1}]$	$[10^{-1},10^{2}]$	1.98	1.96	-0.58	-1.54	-1.79	**8.01**	SN
K_{CB}	$[10^{0},10^{2}]$	$[10^{-3},10^{0}]$	2.54	1.64	**22.14**	-1.95	-1.52	-6.91	PR

a. In square brackets are the intervals of the parameter values, 'm' is the motif group and '~m' the non-motif group. Note that for the kinetic parameters (k_{BC}, k_{CB}, and K_{BC}) showing no apparent bias towards any value classes, the statistical tests were not conducted because their parameter values could not be partitioned into motif group and non-motif group (see Methods).

b. "Pr_m" ("Sn_m") is the mean logarithm value of precision (sensitivity) scores for the motif group, and "$Pr_{\sim m}$" ("$Sn_{\sim m}$") is the same but for the non-motif group.

c. "PR" ("SN") indicates that the corresponding kinetic motif is statistically significant (z-score ≧ 3.29) in improving precision (sensitivity).
